# Supplementary material for: Analysis of PPARγ Signaling Activity in Psoriasis
Source: Int J Mol Sci. 2021 Aug 10;22(16):8603. doi: 10.3390/ijms22168603 (PMC8395241; doi:10.3390/ijms22168603)
Supplement: Supplementary file 1 [file ijms-22-08603-s001.zip › Supplemental materials_Analysis of PPARg signaling activity in psoriasis/Pathway models/Models images and html files/Differentiation of psoriatic T cells/11411.html]

TGFB1


# Protein TGFB1

|  |  |
| --- | --- |
| URN | urn:agi-llid:7040 |
| Total Entities | 1 |
| Connectivity | 20167 |
| Name | TGFB1 |
| Description | TGFB1 |
| Notes | This gene encodes a secreted ligand of the TGF-beta (transforming growth factor-beta) superfamily of proteins. Ligands of this family bind various TGF-beta receptors leading to recruitment and activation of SMAD family transcription factors that regulate gene expression. The encoded preproprotein is proteolytically processed to generate a latency-associated peptide (LAP) and a mature peptide, and is found in either a latent form composed of a mature peptide homodimer, a LAP homodimer, and a latent TGF-beta binding protein, or in an active form consisting solely of the mature peptide homodimer. The mature peptide may also form heterodimers with other TGFB family members. This encoded protein regulates cell proliferation, differentiation and growth, and can modulate expression and activation of other growth factors including interferon gamma and tumor necrosis factor alpha. This gene is frequently upregulated in tumor cells, and mutations in this gene result in Camurati-Engelmann disease. [provided by RefSeq, Aug 2016] |
| Primary Cell Localization | Extracellular |
| Class | Ligand |
| ObjectType | Protein |

---

|  |  |
| --- | --- |
| ChildConcepts | latency-associated peptide |

---

|  |  |
| --- | --- |
| Pathway | Proteins Involved in Stem Cell Exhaustion in Aging |
|  | Prolactin Production |
|  | Cortisol in Resolving Inflammation |
|  | Estrogens in Prolactin Production |
|  | TLR4 Signaling in Leukocytes |
|  | Immunoglobulin Class-Switch Recombination Activation |
|  | Treg-Cell Differentiation |
|  | AHR Signaling in Treg and Dendritic Cells Function |
|  | AHR Signaling in Th17 Cells Function |
|  | Th17-Cell Differentiation |
|  | Plasmin Effects in Inflammation |
|  | Proplatelet Maturation |
|  | Lipoxins and Resolvins in Inflammation Resolution |
|  | Keep out Signal: LTF Inhibits Neutrophil Recruitment and Inflammation |
|  | Eosinophil Survival by Cytokine Signaling |
|  | Eosinophil Activation |
|  | Mast-Cells De Novo Synthesized Mediators via IgE Independent Signaling |
|  | CD16/CD14 Proinflammatory Monocyte Activation |
|  | anti-Inflammatory Function of Macrophage M2 Lineage |
|  | Vascular Endothelial Cell Activation by Growth Factors |
|  | Vascular Smooth Muscle Cell/Pericyte Differentiation and Proliferation |
|  | PRL Expression Regulation by BMP4 and TGFB1 |
|  | G1/S Damage Checkpoint |
|  | Cell Cycle Overiew |
|  | Proteins Involved in Atherosclerosis |
|  | Dilated Cardiomyopathy |
|  | Proteins Involved in Dilated Cardiomyopathy |
|  | Proteins Involved in Hypertrophic Cardiomyopathy |
|  | Cardiomyocyte Hypertrophy |
|  | Hypertrophic Cardiomyopathy |
|  | Proteins Involved in Pulmonary Hypertension |
|  | BMP/TGF-beta Signaling Impairment in Pulmonary Hypertension |
|  | Proteins Involved in Hypoxia-Induced Pulmonary Hypertension |
|  | Myocardial Remodeling |
|  | Myocardial Infarction |
|  | Proteins Involved in Myocardial Ischemia |
|  | Lymphocyte Mediated Myocardial Injury in Myocarditis |
|  | Proteins Involved in Myocarditis |
|  | Hair Follicle Keratinocyte Apoptosis |
|  | Androgens in Sebocyte Maturation |
|  | Proteins Involved in Androgenetic Alopecia |
|  | Androgens Promote Scalp Dermal Papilla Regression |
|  | Proteins with Altered Expression in Androgenetic Alopecia |
|  | Proteins with Altered Expression in Melanoma |
|  | Melanoma |
|  | Proteins Involved in Psoriasis |
|  | T-Cells Differentiation Block in Psoriasis |
|  | Dendritic Cells Function in Psoriasis |
|  | TGF-beta Signaling in Skin Aging |
|  | Proteins Involved in Systemic Lupus Erythematosus |
|  | Proteins Involved in Vitiligo |
|  | Treg-Cell Function in Diabetes Mellitus Type 1 |
|  | Natural Killer T-Cell Roles in Diabetes Mellitus Type 1 |
|  | non-Suppressive Treg-Cell in Diabetes Mellitus Type 1 |
|  | TLR2 Signaling in Treg-Cell in Type 1 Diabetes (Animal Model) |
|  | Proteins Involved in Diabetes Mellitus Type 1 |
|  | Treg-Cell Activation in Diabetes Mellitus |
|  | Proteins Involved in Diabetes Mellitus Type 2 |
|  | Hexosamine Pathway in Diabetic Microangiopathy |
|  | Protein Kinase C in Diabetic Microangiopathy |
|  | Peripheral Tissue Microangiopathy |
|  | Tubular Cell Dysfunction in Progressive Diabetic Nephropathy |
|  | Endothelial Cell Dysfunction in Progressive Diabetic Nephropathy |
|  | Proteins Involved in Diabetic Nephropathy |
|  | Mesangial Cell Dysfunction in Diabetic Nephropathy |
|  | Microangiopathy in Diabetic Neuropathy |
|  | Proliferative Diabetic Retinopathy |
|  | Hepatic Stellate Cells in non-Alcoholic Fatty Liver Disease |
|  | Proteins Involved in non-Alcoholic Fatty Liver Disease |
|  | Immune Sustem Activation in Hashimoto's Thyroiditis |
|  | Proteins Involved in Hashimoto's Thyroiditis |
|  | Hashimoto's Thyroiditis |
|  | Ethanol Induced Efects in Hyperprolactinemia |
|  | Graves Ophthalmopathy |
|  | Proteins Involved in Myasthenia Gravis |
|  | Proteins Involved in Obesity |
|  | Proteins Involved in Prolactinoma |
|  | Proteins Involved in Celiac Disease |
|  | Proteins Involved in Colorectal Neoplasms |
|  | Metastatic Colorectal Cancer |
|  | GUCY2C Signaling in Colorectal Cancer |
|  | Crohn's Disease |
|  | Th17-Cell Activation in Crohn's Disease |
|  | Proteins Involved in Inflammatory Bowel Diseases |
|  | Dendritic Cell Dysfunction in Crohn's disease |
|  | Proteins Involved in Ulcerative Colitis |
|  | Chronic Myeloid Leukemia |
|  | Diffuse Large-B-Cell Lymphoma |
|  | Proteins Involved in Diffuse Large-B-Cell Lymphoma |
|  | Proteins Involved in Follicular Lymphoma |
|  | Hodgkin and Reed-Sternberg Cells in Hodgkin Lymphoma |
|  | Proteins Involved in Hodgkin Lymphoma |
|  | Hodgkin Lymphoma |
|  | Proteins Involved in Multiple Myeloma |
|  | Proteins Involved in T-Cell Acute Lymphoblastic Leukemia |
|  | SERPINA1 Associated Pulmonary Emphysema |
|  | Vascularization in Hepatocellular Carcinoma |
|  | Proteins Involved in Hepatocellular Carcinoma |
|  | Hepatocellular Carcinoma |
|  | Growth Factor Signaling in Hepatocellular Carcinoma |
|  | Proteins Involved in Pancreatic Cancer |
|  | Pancreatic Ductal Carcinoma |
|  | Pancreatic Neuroendocrine Tumors |
|  | TGFBR Signaling in Pancreatic Neoplasms |
|  | ADAM33 Role in Asthma |
|  | Eosinophil Survival in Asthma |
|  | Th17-Cell Differentiation in Asthma |
|  | Proteins Involved in Cystic Fibrosis |
|  | Proteins Involved in Osteoarthritis |
|  | Dendritic Cell Dysfunction in Psoriatic Arthritis |
|  | Th17-Cell and Th1 Immune Responsein Psoriatic Arthritis |
|  | Synovial Fibroblast Proliferation in Rheumatoid Arthritis |
|  | Proteins Involved in Rheumatoid Arthritis |
|  | Autocrine Cytokine/Chemokine Loops in Systemic Scleroderma |
|  | Th2-Cells Function in Systemic Scleroderma |
|  | Genes with Mutations Associated with Systemic Scleroderma |
|  | Proteins Involved in Systemic Scleroderma |
|  | Skin Fibrosis |
|  | Proteins with Altered Expression in Systemic Scleroderma |
|  | Proteins Involved in HIV Type 1 Infection |
|  | Th17-Cell Numbers Reduction in HIV |
|  | CD4+ T-Cell Function Decline in HIV |
|  | Proteins Involved in Periodontitis |
|  | Hereditary Hemorrhagic Telangiectasia |
|  | Proteins Involved in Hereditary Hemorrhagic Telangiectasia |
|  | Proteins Involved in Autistic Disorder |
|  | Proteins Involved in Autistic Disorder and Aggression |
|  | Proteins Involved in Hutchinson-Gilford Progeria Sindrome |
|  | Muscular Dystrophies |
|  | Proteins Involved in Osteoporosis |
|  | Proteins with Altered Expression in Rheumatoid Arthritis |
|  | Proteins Involved in Alzheimer's Disease |
|  | Proteins Involved in Glioblastoma |
|  | Proteins Involved in Ependymoma |
|  | Proteins Involved in Glioma |
|  | Glioma Stem Cell Program Activation |
|  | Medulloblastoma |
|  | Proteins Involved in Medulloblastoma |
|  | Proteins Involved in Migraine |
|  | Proteins Involved in Amyotrophic Lateral Sclerosis |
|  | Proteins Involved in Multiple Sclerosis |
|  | IL6/IL12 Signaling Activates Immune System in Multiple Sclerosis |
|  | Breast Cancer |
|  | Proteins Involved in Endometriosis |
|  | Proteins Involved in Spontaneous Abortion |
|  | Ovarian Cancer |
|  | Proteins with Altered Expression in Ovarian Cancer |
|  | Proteins Involved in Cholesteatoma |
|  | Proteins Involved in HPV Infection |
|  | Prostate Cancer |
|  | Proteins Involved in Prostate Cancer |
|  | Proteins Involved in Age-Related Macular Degeneration |
|  | Proteins Involved in Cataract |
|  | Steroids Induced Cataract |
|  | MYOC Associated Glaucoma |
|  | Proteins Involved in Glaucoma |
|  | Cochlear Inflammation (Noise-Induced) |
|  | Proteins Involved in Otitis Media |
|  | Cytokines Trigger Otitis Media |
|  | Immune System Role in Otitis Media |
|  | Alveolar Macrophages Dysfunction in COPD |
|  | Proteins Involved in Chronic Obstructive Pulmonary Disease |
|  | Alveolar Epithelial Cell Dysfunction in COPD |
|  | Proteins Involved in Pulmonary Emphysema |
|  | Proteins Involved in Chronic Bronchitis |
|  | Genes with Mutations Associated with Cystic Fibrosis |
|  | Advanced Glycation End Products in Diabetes or Age Related Erectile Dysfunction |
|  | Proteins with Altered Expression in Erectile Dysfunction |
|  | Proteins Involved in Erectile Dysfunction |
|  | Proteins Involved in Glomerulonephritis |
|  | Proteins with Altered Expression in Glomerulonephritis |
|  | Podocyte Dysfunction in Glomerulonephritis |
|  | Mesangial Cell Dysfunction in Glomerulonephritis |
|  | Interstitial Fibroblasts in Pyelonephritis |
|  | Proteins with Altered Expression in Pyelonephritis |
|  | Proteins Involved in Cryptorchidism |
|  | S100B Expression Targets |
|  | Dopamine/Gi Expression Targets |
|  | BMP2/BMPR2 Expression Targets |
|  | BMP7/ACVR2 Expression Targets |
|  | BMP4/BMPR2 Expression Targets |
|  | BMP7/BMPR2/ACVR2 Expression Targets |
|  | TGFBR3 -> ARRB2 Signaling |
|  | CD19 Expression Targets |
|  | CD86 -> ATF/CREB/CREBBP Expression Targets |
|  | CD86 -> STAT Expression Targets |
|  | CD86 -> NF-kB Expression Targets |
|  | Elevated Receptors -> Expression Targets in Blood vessel |
|  | Elevated Receptors -> Expression Targets in Blood Vessel |
|  | CCL3L1 Expression Target |
|  | CCL15 Expression Targets |
|  | CSF3 Expression Targets |
|  | CSF2 -> STAT Expression Targets |
|  | CNTF Expression Targets |
|  | Ethanol Induced Hepatotoxicity |
|  | Cyclosporine Induced Nephrotoxicity |
|  | Elevated Receptors -> Expression Targets in Pancreas |
|  | F2 -> AP-1/CREB/ELK/SRF/SP1 Expression Targets |
|  | F2 -> STAT1/NF-kB Expression Targets |
|  | FGF9 -> AP-1/CREB/MYC Expression Targets |
|  | FGF2 -> STAT Expression Targets |
|  | FGF9 -> STAT Expression Targets |
|  | FGF2 -> AP-1/CREB/CREBBP/ELK/SRF/MYC Expression Targets |
|  | GCG Expression Targets |
|  | Glutamate/Gq Expression Targets |
|  | VIP Expression Targets |
|  | HGF -> AP-1/CREB/ELK/SRF/MYC Expression Targets |
|  | DNA Damage Checkpoint Impairment in Cancer |
|  | IL23A and IL17A Provoke Cancer-Associated Inflammation |
|  | STAT3 Facilitates the Function of Treg Cells and Cancer Progression |
|  | IL12 Antitumor Effect is Blocked in Cancer |
|  | Proteins with Altered Expression in Tumor-Promoting Inflammation |
|  | N2 Neutrophils in Tumor-Promoting Inflammation and Tumor Progression |
|  | TGFB Signaling activation by Blocking of Tumor Suppressors |
|  | HIF1A in Vasculogenic Mimicry of Cancer |
|  | VEGFA Dependent Angiogenesis in Cancer |
|  | Inhibition of THBS1 Activates Angiogenesis in Cancer |
|  | FBLN5 Potentiates Migration and Proliferation of Endothelial Cells in Cancer |
|  | Integrins in Cancer Cell Motility, Invasion and Survival |
|  | Proteins with Altered Expression in Cancer Metastases |
|  | TGFB Family in Epithelial to Mesenchymal Transition in Cancer |
|  | Invadopodia Formation in Cancer Cells |
|  | Treg Cells Promote Immunosuppression in Cancer Immune Escape |
|  | Tumor Infiltrating Macrophages in Cancer Progression and Immune Escape |
|  | MHC1 Causes Antigen Presentation Failure in Cancer Immune Escape |
|  | Myeloid Derived Suppressor Cells in Cancer Immune Escape |
|  | INHBA/ACVR2/BMPR Expression Targets |
|  | INHBA/ACVR2/ACVR1 Expression Targets |
|  | ICAM1 Expression Targets |
|  | Fibrinogen Expression Targets |
|  | NOTCH -> Expression Targets in Skin |
|  | IL13 Expression Targets |
|  | IL3 Expression Targets |
|  | IL5 Expression Targets |
|  | IL9 Expression Targets |
|  | Leptin -> STAT Expression Targets |
|  | Leptin -> ELK/SRF Expression Targets |
|  | Prostaglandin F Expression Targets |
|  | LPA Expression Targets |
|  | PAF/Gq -> AP-1/ATF1/CREB/ERK/SRF Expression Targets |
|  | Dronabinol/Anandamide Expression Targets |
|  | TNF Receptors -> Expression Targets in Lymphoid System and Blood |
|  | CD40 -> Expression Targets in Thymus |
|  | CD40 -> Expression Targets in Lymph Node |
|  | Elevated Receptors -> Expression Targets in Thymus |
|  | P2RX7 -> Expression Targets in Nerve Tissue |
|  | NGF -> SMAD3/NF-kB Expression Targets |
|  | NGF -> AP-1/TP53/MYC Expression Targets |
|  | NTF3 Expression Targets |
|  | Adenosine Expression Targets |
|  | PDGF -> AP-1/CREB/CREBBP/MYC Expression Targets |
|  | PDGF -> STAT Expression Targets |
|  | AVP/Gs -> CREB/ELK/SRF/AP-1/EGR Expression Targets |
|  | GNRH1 Expression Targets |
|  | POMC Expression Targets |
|  | AGT -> CREB Expression Targets |
|  | NPY Expression Targets |
|  | AVP/Gq -> STAT Expression Targets |
|  | TAC1 Expression Targets |
|  | AVP/Gs -> STAT Expression Targets |
|  | AGT -> STAT Expression Targets |
|  | AVP/Gq -> CREB/ELK/SRF/AP-1/EGR Expression Targets |
|  | MDK/PTN Expression Targets |
|  | PAF Expression Targets |
|  | TGFBR -> AP-1 Signaling |
|  | TGFBR/BMPR -> SMAD2/3 Signaling |
|  | TGFBR -> CREB/ELK-SRF Signaling |
|  | TGFBR -> ATF/GADD/MAX/TP53 Signaling |
|  | TGFBR -> SMAD1/5/9 Signaling |
|  | TGFBR -> MEF/MYOD/MYOG Signaling |
|  | TGFBR1/AP-1 Signaling |
|  | MSTN/ACVR2/ACVR1 Expression Targets |
|  | NODAL/ACVR2B Expression Targets |
|  | Elevated Receptors -> Expression Targets in Lung |
|  | TGF-beta Signaling |
|  | TCR -> NF-kB Expression Targets |
|  | TCR -> STAT Expression Targets |
|  | TCR -> AP-1 Expression Targets |
|  | TGFA -> STAT Expression Targets |
|  | TGFA -> CTNNB/CTNND Expression Targets |
|  | TGFA -> HIF1A Expression Targets |
|  | TGFA/AP-1/ATF Expression Targets |
|  | TGFA -> CREB/CREBBP/ELK-SRF/MYC Expression Targets |
|  | TGFB1-TGFBR1 Expression Targets |
|  | TGFB3-TGFBR2 Expression Targets |
|  | TGFB2-TGFBR2 Expression Targets |
|  | TGFB1-TGFBR2 Expression Targets |
|  | TGFB1-TGFBR1/AP-1 Expression Targets |
|  | TGFB1-ACVRL1 Expression Targets |
|  | TNF -> NF-kB Expression Targets |
|  | TNF -> AP-1 Expression Targets |
|  | TNF -> STAT Expression Targets |
|  | Thrombopoietin -> AP-1/CREB/CREBBP/MYC Expression Targets |
|  | Thrombopoietin -> STAT Expression Targets |
|  | TLR7 Expression Targets |
|  | IL1B -> PGE2 Expression Targets |
|  | IL1B Expression Targets |
|  | TLR4 -> NF-kB/IRF Expression Targets |
|  | IL1A Expression Targets |
|  | Elevated Receptors -> Expression Targets in Kidney |
|  | cypa |
|  | PPAR Psoriasis |
|  | prarg neg,ukn expres targets, ps-positive |
|  | PPARG negative targets |
|  | pparg expr target\_regulators |
|  | Neighbors of disease exacerbation |
|  | Neighbors of tumor progression |
|  | Neighbors of cancer progression |
|  | Neighbors of inflammatory status |
|  | Neighbors of overall survival |
|  | Neighbors of disease severity |
|  | Neighbors of tumor size |
|  | Neighbors of lesion size |
|  | Neighbors of mortality |
|  | Neighbors of survival rate |
|  | Neighbors of infarct size |
|  | Neighbors of survival time |
|  | Neighbors of functional recovery |
|  | Neighbors of insulin sensitivity |
|  | Neighbors of bacterial load |
|  | Neighbors of cancer survival |
|  | Neighbors of graft survival |
|  | Neighbors of therapeutic efficacy |
|  | Neighbors of clinical stage |
|  | Neighbors of lethality |
|  | Neighbors of tumor regression |
|  | Neighbors of recurrence-free survival |
|  | Neighbors of disease-free survival |
|  | Neighbors of tumor response |
|  | Neighbors of macrophage count |
|  | Neighbors of long-term survival |
|  | Neighbors of treatment outcome |
|  | Neighbors of bone mass |
|  | Neighbors of body weight |
|  | Neighbors of body weight gain |
|  | Neighbors of cardiovascular risk |
|  | Neighbors of microvessel density |
|  | Neighbors of tumor recurrence |
|  | Neighbors of tumor promotion |
|  | Neighbors of neutrophil count |
|  | Neighbors of glucose tolerance |
|  | Neighbors of morbidity |
|  | Neighbors of fibrosis progression |
|  | Neighbors of mortality rate |
|  | Neighbors of fat mass |
|  | Neighbors of exhaustion |
|  | Neighbors of bone mineral density |
|  | Neighbors of adverse outcome |
|  | Neighbors of radioresistance |
|  | Neighbors of lesion area |
|  | Neighbors of cell density |
|  | Neighbors of cancer-specific survival |
|  | Neighbors of progression-free survival |
|  | Neighbors of apoptosis rate |
|  | Neighbors of early mortality |
|  | Neighbors of shear stress |
|  | Neighbors of viral load |
|  | Neighbors of blood pressure |
|  | Neighbors of leukocyte count |
|  | Neighbors of tumor grade |
|  | Neighbors of vascular reactivity |
|  | Neighbors of vascular leakage |
|  | Neighbors of body mass index |
|  | Neighbors of vascular density |
|  | Neighbors of muscle mass |
|  | Neighbors of cancer risk |
|  | Neighbors of cancer incidence |
|  | Neighbors of tumor aggressiveness |
|  | Neighbors of systolic blood pressure |
|  | Neighbors of tumor resistance |
|  | Neighbors of blood glucose |
|  | Neighbors of ejection fraction |
|  | Neighbors of renal hypertrophy |
|  | Neighbors of fasting plasma glucose |
|  | Neighbors of osteoclast number |
|  | Neighbors of cancer recurrence |
|  | Neighbors of T-cell count |
|  | Neighbors of antimetastatic activity |
|  | Neighbors of glucose level |
|  | Neighbors of vascular tone |
|  | Neighbors of radiosensitivity |
|  | Neighbors of breast cancer risk |
|  | Neighbors of proliferation index |
|  | Neighbors of cell permeability |
|  | Neighbors of pregnancy outcome |
|  | Neighbors of functional status |
|  | Neighbors of gastric cancer risk |
|  | Neighbors of neurological outcome |
|  | Neighbors of infarct area |
|  | Neighbors of fertility |
|  | Neighbors of pathological response |
|  | Neighbors of lymphocyte count |
|  | Neighbors of skin thickness |
|  | Neighbors of metabolic response |
|  | Neighbors of insulin level |
|  | Neighbors of comorbidity |
|  | Neighbors of carotid intima-media thickness |
|  | Neighbors of bone volume |
|  | Neighbors of fibroblast phenotype |
|  | Neighbors of fractional shortening |
|  | Neighbors of disease exacerbation |
|  | Neighbors of tumor progression |
|  | Neighbors of cancer progression |
|  | Neighbors of inflammatory status |
|  | Neighbors of overall survival |
|  | Neighbors of disease severity |
|  | Neighbors of tumor size |
|  | Neighbors of lesion size |
|  | Neighbors of mortality |
|  | Neighbors of survival rate |
|  | Neighbors of infarct size |
|  | Neighbors of survival time |
|  | Neighbors of functional recovery |
|  | Neighbors of insulin sensitivity |
|  | Neighbors of bacterial load |
|  | Neighbors of cancer survival |
|  | Neighbors of graft survival |
|  | Neighbors of therapeutic efficacy |
|  | Neighbors of clinical stage |
|  | Neighbors of lethality |
|  | Neighbors of tumor regression |
|  | Neighbors of recurrence-free survival |
|  | Neighbors of disease-free survival |
|  | Neighbors of tumor response |
|  | Neighbors of macrophage count |
|  | Neighbors of long-term survival |
|  | Neighbors of treatment outcome |
|  | Neighbors of bone mass |
|  | Neighbors of body weight |
|  | Neighbors of body weight gain |
|  | Neighbors of cardiovascular risk |
|  | Neighbors of microvessel density |
|  | Neighbors of tumor recurrence |
|  | Neighbors of tumor promotion |
|  | Neighbors of neutrophil count |
|  | Neighbors of glucose tolerance |
|  | Neighbors of morbidity |
|  | Neighbors of fibrosis progression |
|  | Neighbors of mortality rate |
|  | Neighbors of fat mass |
|  | Neighbors of exhaustion |
|  | Neighbors of bone mineral density |
|  | Neighbors of adverse outcome |
|  | Neighbors of radioresistance |
|  | Neighbors of lesion area |
|  | Neighbors of cell density |
|  | Neighbors of cancer-specific survival |
|  | Neighbors of progression-free survival |
|  | Neighbors of apoptosis rate |
|  | Neighbors of early mortality |
|  | Neighbors of shear stress |
|  | Neighbors of viral load |
|  | Neighbors of blood pressure |
|  | Neighbors of leukocyte count |
|  | Neighbors of tumor grade |
|  | Neighbors of vascular reactivity |
|  | Neighbors of vascular leakage |
|  | Neighbors of body mass index |
|  | Neighbors of vascular density |
|  | Neighbors of muscle mass |
|  | Neighbors of cancer risk |
|  | Neighbors of cancer incidence |
|  | Neighbors of tumor aggressiveness |
|  | Neighbors of systolic blood pressure |
|  | Neighbors of tumor resistance |
|  | Neighbors of blood glucose |
|  | Neighbors of ejection fraction |
|  | Neighbors of renal hypertrophy |
|  | Neighbors of fasting plasma glucose |
|  | Neighbors of osteoclast number |
|  | Neighbors of cancer recurrence |
|  | Neighbors of T-cell count |
|  | Neighbors of antimetastatic activity |
|  | Neighbors of glucose level |
|  | Neighbors of vascular tone |
|  | Neighbors of radiosensitivity |
|  | Neighbors of breast cancer risk |
|  | Neighbors of proliferation index |
|  | Neighbors of cell permeability |
|  | Neighbors of pregnancy outcome |
|  | Neighbors of functional status |
|  | Neighbors of gastric cancer risk |
|  | Neighbors of neurological outcome |
|  | Neighbors of infarct area |
|  | Neighbors of fertility |
|  | Neighbors of pathological response |
|  | Neighbors of lymphocyte count |
|  | Neighbors of skin thickness |
|  | Neighbors of metabolic response |
|  | Neighbors of insulin level |
|  | Neighbors of comorbidity |
|  | Neighbors of carotid intima-media thickness |
|  | Neighbors of bone volume |
|  | Neighbors of fibroblast phenotype |
|  | Neighbors of fractional shortening |
|  | Neighbors of cell development |
|  | Neighbors of immune response |
|  | Neighbors of cell population |
|  | Neighbors of T-cell development |
|  | Neighbors of inflammatory response |
|  | Neighbors of wound healing |
|  | Neighbors of angiogenesis |
|  | Neighbors of innate immune response |
|  | Neighbors of cell function |
|  | Neighbors of macrophage activation |
|  | Neighbors of cell formation |
|  | Neighbors of adaptive immune response |
|  | Neighbors of osteoclast development |
|  | Neighbors of epithelial to mesenchymal transition |
|  | Neighbors of immunity |
|  | Neighbors of cell proliferative response |
|  | Neighbors of chemotaxis |
|  | Neighbors of dendritic cell differentiation |
|  | Neighbors of cell survival |
|  | Neighbors of osteoclast differentiation |
|  | Neighbors of cell phenotype |
|  | Neighbors of T-cell proliferation |
|  | Neighbors of tissue remodeling |
|  | Neighbors of cell adhesion |
|  | Neighbors of T-cell activation |
|  | Neighbors of endothelial cell proliferation |
|  | Neighbors of fibrogenesis |
|  | Neighbors of tumor growth |
|  | Neighbors of dendritic cell development |
|  | Neighbors of tissue repair |
|  | Neighbors of cell count |
|  | Neighbors of cell infiltration |
|  | Neighbors of cell interaction |
|  | Neighbors of SMC proliferation |
|  | Neighbors of lumen formation |
|  | Neighbors of endothelial cell migration |
|  | Neighbors of adipogenesis |
|  | Neighbors of lipid storage |
|  | Neighbors of cell damage |
|  | Neighbors of pregnancy |
|  | Neighbors of monocyte differentiation |
|  | Neighbors of aging |
|  | Neighbors of cell motility |
|  | Neighbors of bone resorption |
|  | Neighbors of monocyte migration |
|  | Neighbors of fibroblast proliferation |
|  | Neighbors of adipocyte differentiation |
|  | Neighbors of macrophage migration |
|  | Neighbors of microglial activation |
|  | Neighbors of neutrophil recruitment |
|  | Neighbors of regeneration |
|  | Neighbors of cell migration |
|  | Neighbors of cancer growth |
|  | Neighbors of stem cell proliferation |
|  | Neighbors of smooth muscle cell migration |
|  | Neighbors of hepatic regeneration |
|  | Neighbors of transendothelial migration |
|  | Neighbors of phagocytosis |
|  | Neighbors of immune system activation |
|  | Neighbors of leukocyte migration |
|  | Neighbors of leukocyte recruitment |
|  | Neighbors of osteoclast formation |
|  | Neighbors of monocyte recruitment |
|  | Neighbors of cell invasion |
|  | Neighbors of heart function |
|  | Neighbors of hemopoiesis |
|  | Neighbors of bone remodeling |
|  | Neighbors of ossification |
|  | Neighbors of T-cell function |
|  | Neighbors of immune system function |
|  | Neighbors of lung development |
|  | Neighbors of autophagy |
|  | Neighbors of monocyte adhesion |
|  | Neighbors of transcription activation |
|  | Neighbors of senescence |
|  | Neighbors of T-cell response |
|  | Neighbors of tumor immunity |
|  | Neighbors of hepatocyte proliferation |
|  | Neighbors of cell transdifferentiation |
|  | Neighbors of neuroprotection |
|  | Neighbors of stem cell differentiation |
|  | Neighbors of endothelial cell function |
|  | Neighbors of neuronal death |
|  | Neighbors of cell growth |
|  | Neighbors of ROS generation |
|  | Neighbors of epithelial cell proliferation |
|  | Neighbors of first trimester pregnancy |
|  | Neighbors of cancer cell growth |
|  | Neighbors of sensitization |
|  | Neighbors of hemato-encephalic barrier |
|  | Neighbors of macrophage function |
|  | Neighbors of cellular immune response |
|  | Neighbors of cellular senescence |
|  | Neighbors of colony formation |
|  | Neighbors of macrophage differentiation |
|  | Neighbors of cell differentiation |
|  | Neighbors of keratinocyte proliferation |
|  | Neighbors of stem cell migration |
|  | Neighbors of life span |
|  | Neighbors of cell homeostasis |
|  | Neighbors of psoriasis |
|  | Neighbors of inflammatory disease |
|  | Neighbors of rheumatoid arthritis |
|  | Neighbors of atherosclerosis |
|  | Neighbors of fibrosis |
|  | Neighbors of colitis |
|  | Neighbors of arthritis |
|  | Neighbors of inflammatory bowel disease |
|  | Neighbors of injury |
|  | Neighbors of atherogenesis |
|  | Neighbors of vascular remodeling |
|  | Neighbors of metastasis |
|  | Neighbors of carcinogenesis |
|  | Neighbors of multiple sclerosis |
|  | Neighbors of chronic inflammation |
|  | Neighbors of inflammation |
|  | Neighbors of tumor microenvironment |
|  | Neighbors of pathological angiogenesis |
|  | Neighbors of obesity |
|  | Neighbors of vascular disease |
|  | Neighbors of diabetes mellitus |
|  | Neighbors of asthma |
|  | Neighbors of pneumonia |
|  | Neighbors of hyperplasia |
|  | Neighbors of osteoarthritis |
|  | Neighbors of liver fibrosis |
|  | Neighbors of chronic obstructive pulmonary disease |
|  | Neighbors of myocardial infarction |
|  | Neighbors of leukocyte infiltration |
|  | Neighbors of reperfusion injury |
|  | Neighbors of airway inflammation |
|  | Neighbors of hepatitis |
|  | Neighbors of insulin resistance |
|  | Neighbors of infection |
|  | Neighbors of vascular injury |
|  | Neighbors of renal injury |
|  | Neighbors of preeclampsia |
|  | Neighbors of macrophage infiltration |
|  | Neighbors of liver injury |
|  | Neighbors of neuroinflammation |
|  | Neighbors of sepsis |
|  | Neighbors of death |
|  | Neighbors of type 1 diabetes |
|  | Neighbors of encephalomyelitis |
|  | Neighbors of autoimmune disease |
|  | Neighbors of ischemic stroke |
|  | Neighbors of dermatitis |
|  | Neighbors of neutrophil infiltration |
|  | Neighbors of heart disease |
|  | Neighbors of diabetic nephropathy |
|  | Neighbors of lung injury |
|  | Neighbors of immunopathology |
|  | Neighbors of systemic sclerosis |
|  | Neighbors of hepatocellular carcinoma |
|  | Neighbors of autoimmunity |
|  | Neighbors of acute lung injury |
|  | Neighbors of gastric cancer |
|  | Neighbors of atherosclerotic plaque |
|  | Neighbors of experimental autoimmune encephalomyelitis |
|  | Neighbors of colorectal cancer |
|  | Neighbors of cardiac remodeling |
|  | Neighbors of breast cancer |
|  | Neighbors of ischemia |
|  | Neighbors of systemic lupus erythematosus |
|  | Neighbors of melanoma |
|  | Neighbors of collagen-induced arthritis |
|  | Neighbors of cartilage degeneration |
|  | Neighbors of interstitial fibrosis |
|  | Neighbors of neoplasm invasion |
|  | Neighbors of kidney disease |
|  | Neighbors of heart failure |
|  | Neighbors of lung disease |
|  | Neighbors of pulmonary fibrosis |
|  | Neighbors of cardiovascular disease |
|  | Neighbors of stroke |
|  | Neighbors of cancer |
|  | Neighbors of cerebral ischemia |
|  | Neighbors of type 2 diabetes |
|  | Neighbors of neoplasm |
|  | Neighbors of endometriosis |
|  | Neighbors of neointima |
|  | Neighbors of vasculitis |
|  | Neighbors of atrophy |
|  | Neighbors of diet-induced obesity |
|  | Neighbors of respiratory hypersensitivity |
|  | Neighbors of myocardial fibrosis |
|  | Neighbors of synovitis |
|  | Neighbors of coronary artery disease |
|  | Neighbors of rupture |
|  | Neighbors of endothelial cell dysfunction |
|  | Neighbors of liver disease |
|  | Neighbors of prostate cancer |
|  | Neighbors of hypertrophy |
|  | Neighbors of osteolysis |
|  | Neighbors of ovarian cancer |
|  | Neighbors of pancreatic cancer |
|  | Neighbors of hypertension |
|  | Neighbors of intimal hyperplasia |
|  | Neighbors of allograft rejection |
|  | Neighbors of proteinuria |
|  | Neighbors of epithelial cell |
|  | Neighbors of keratinocyte |
|  | Neighbors of vascular smooth muscle cell |
|  | Neighbors of umbilical vein endothelial cell |
|  | Neighbors of monocyte |
|  | Neighbors of endometrial stromal cell |
|  | Neighbors of alveolar macrophage |
|  | Neighbors of synoviocyte |
|  | Neighbors of macrophage |
|  | Neighbors of adipocyte |
|  | Neighbors of activated monocyte |
|  | Neighbors of fibroblast |
|  | Neighbors of airway epithelial cell |
|  | Neighbors of PBMC |
|  | Neighbors of stromal cell |
|  | Neighbors of airway smooth muscle cell |
|  | Neighbors of osteoblast |
|  | Neighbors of pneumocyte |
|  | Neighbors of endometrium cell |
|  | Neighbors of microglia |
|  | Neighbors of trophoblast |
|  | Neighbors of intestine epithelium cell |
|  | Neighbors of mononuclear cell |
|  | Neighbors of hepatic stellate cell |
|  | Neighbors of hepatocyte |
|  | Neighbors of decidual cell |
|  | Neighbors of kidney mesangial cell |
|  | Neighbors of endothelial cell |
|  | Neighbors of dendritic cell |
|  | Neighbors of Kuppfer cell |
|  | Neighbors of proadipocyte |
|  | Neighbors of kidney tubule cell |
|  | Neighbors of inflammatory cell |
|  | Neighbors of bone marrow macrophage |
|  | Neighbors of peritoneal macrophage |
|  | Neighbors of osteoclast |
|  | Neighbors of neutrophil |
|  | Neighbors of smooth muscle myocyte |
|  | Neighbors of astrocyte |
|  | Neighbors of bone marrow derived dendritic cell |
|  | Neighbors of lung fibroblast |
|  | Neighbors of activated T-cell |
|  | Neighbors of mesothelial cell |
|  | Neighbors of mononuclear phagocyte |
|  | Neighbors of capillary endothelial cell |
|  | Neighbors of immunocompetent cell |
|  | Neighbors of granulocyte |
|  | Neighbors of cardiac myocyte |
|  | Neighbors of cancer-associated fibroblast |
|  | Neighbors of myeloid cell |
|  | Neighbors of mesenchymal stroma cell |
|  | Neighbors of corneal epithelial cell |
|  | Neighbors of cytotrophoblast |
|  | Neighbors of stem cell |
|  | Neighbors of tumor infiltrating macrophage |
|  | Neighbors of sinusoidal cell |
|  | Neighbors of B-cell |
|  | Neighbors of granulosa cell |
|  | Neighbors of leukocyte |
|  | Neighbors of chondrocyte |
|  | Neighbors of insulin-secreting cell |
|  | Neighbors of mesenchymal cell |
|  | Neighbors of T-cell |
|  | Neighbors of M1 macrophage |
|  | Neighbors of myofibroblast |
|  | Neighbors of pancreatic stellate cell |
|  | Neighbors of mast cell |
|  | Neighbors of bone marrow stromal cell |
|  | Neighbors of spleen cell |
|  | Neighbors of brain capillary endothelial cell |
|  | Neighbors of neuroglia |
|  | Neighbors of thyroid epithelial cell |
|  | Neighbors of aortic smooth muscle cell |
|  | Neighbors of hematopoietic cell |
|  | Neighbors of podocyte |
|  | Neighbors of mammary epithelial cell |
|  | Neighbors of conventional dendritic cell |
|  | Neighbors of pericyte |
|  | Neighbors of antigen-presenting cell |
|  | Neighbors of epidermis cell |
|  | Neighbors of bone marrow derived mast cell |
|  | Neighbors of phagocyte |
|  | Neighbors of kidney cell |
|  | Neighbors of syncytiotrophoblast |
|  | Neighbors of lymphocyte |
|  | Neighbors of Langerhans cell |
|  | Neighbors of thymic epithelial cell |
|  | Neighbors of brain cell |
|  | Neighbors of M2 macrophage |
|  | Neighbors of peritoneum cell |
|  | Neighbors of lymphoid cell |
|  | Neighbors of retina cell |
|  | Neighbors of blood cell |
|  | Neighbors of skin cell |
|  | Neighbors of luteal cell |
|  | Neighbors of peripheral lymphocyte |
|  | Neighbors of activated B-cell |
|  | Neighbors of lymph node cell |
|  | Neighbors of lung |
|  | Neighbors of liver |
|  | Neighbors of kidney |
|  | Neighbors of skin |
|  | Neighbors of intestine |
|  | Neighbors of lymph node |
|  | Neighbors of colon |
|  | Neighbors of airway |
|  | Neighbors of blood vessel |
|  | Neighbors of heart |
|  | Neighbors of aorta |
|  | Neighbors of gastrointestinal tract |
|  | Neighbors of microvessel |
|  | Neighbors of bone |
|  | Neighbors of placenta |
|  | Neighbors of carotid artery |
|  | Neighbors of synovial membrane |
|  | Neighbors of uterus |
|  | Neighbors of esophagus |
|  | Neighbors of periodontium |
|  | Neighbors of retina |
|  | Neighbors of brain |
|  | Neighbors of ovary |
|  | Neighbors of breast |
|  | Neighbors of stomach |
|  | Neighbors of islets of Langerhans |
|  | Neighbors of joint |
|  | Neighbors of artery |
|  | Neighbors of cardiovascular system |
|  | Neighbors of pancreas |
|  | Neighbors of prostate |
|  | Neighbors of central nervous system |
|  | Neighbors of kidney glomerulus |
|  | Neighbors of lymphatic system |
|  | Neighbors of colorectal region |
|  | Neighbors of decidua |
|  | Neighbors of amnion |
|  | Neighbors of immune system |
|  | Neighbors of spleen |
|  | Neighbors of peritoneum |
|  | Neighbors of allantois |
|  | Neighbors of alveolus |
|  | Neighbors of coronary artery |
|  | Neighbors of left ventricle |
|  | Neighbors of uterine cervix |
|  | Neighbors of omentum |
|  | Neighbors of arteriole |
|  | Neighbors of bronchus |
|  | Neighbors of glands |
|  | Neighbors of nose |
|  | Neighbors of eye |
|  | Neighbors of cornea |
|  | Neighbors of hippocampus |
|  | Neighbors of intervertebral disc |
|  | Neighbors of lymph node of mesentery |
|  | Neighbors of mammary gland |
|  | Neighbors of vagina |
|  | Neighbors of vitreous body |
|  | Neighbors of cerebral cortex |
|  | Neighbors of bladder |
|  | Neighbors of draining lymph node |
|  | Neighbors of nervous system |
|  | Neighbors of blastocyst |
|  | Neighbors of nasopharynx |
|  | Neighbors of periodontal ligament |
|  | Neighbors of trachea |
|  | Neighbors of nerve |
|  | Neighbors of endocrine system |
|  | Neighbors of small intestine |
|  | Neighbors of aggregated lymphoid follicle of small intestine |
|  | Neighbors of lymphatic vessels |
|  | Neighbors of mediastinal lymph node group |
|  | Neighbors of left atrium |
|  | Neighbors of hypothalamus |
|  | Neighbors of choroid plexus |
|  | Neighbors of hair follicle |
|  | Neighbors of NF-kB family |
|  | Neighbors of oxidized LDL |
|  | Neighbors of JNK |
|  | Neighbors of IL1 family |
|  | Neighbors of PPAR |
|  | Neighbors of Jun/Fos |
|  | Neighbors of NFKBI |
|  | Neighbors of PKC |
|  | Neighbors of PI3K |
|  | Neighbors of STAT family |
|  | Neighbors of mitogen-activated protein kinase |
|  | Neighbors of IFNAR ligand |
|  | Neighbors of transforming growth factor |
|  | Neighbors of TLR |
|  | Neighbors of cytokine |
|  | Neighbors of interferon |
|  | Neighbors of interleukin |
|  | Neighbors of JAK |
|  | Neighbors of MEK1/2 |
|  | Neighbors of PDGF |
|  | Neighbors of Ras GTPase |
|  | Neighbors of ERK1/2 |
|  | Neighbors of growth factor |
|  | Neighbors of PKA |
|  | Neighbors of ovalbumin |
|  | Neighbors of LDL |
|  | Neighbors of NF-AT family |
|  | Neighbors of beta adrenoceptor |
|  | Neighbors of chorionic gonadotropin |
|  | Neighbors of adipokine |
|  | Neighbors of protein tyrosine kinase |
|  | Neighbors of NAD(P)H oxidase |
|  | Neighbors of IgG |
|  | Neighbors of SMAD subfamily |
|  | Neighbors of AMPK |
|  | Neighbors of LXR |
|  | Neighbors of HDL |
|  | Neighbors of inflammatory cytokine |
|  | Neighbors of IL23 |
|  | Neighbors of estrogen receptor |
|  | Neighbors of T-cell receptor |
|  | Neighbors of histone deacetylase |
|  | Neighbors of PDGF-BB |
|  | Neighbors of HIF-1 |
|  | Neighbors of IL12 |
|  | Neighbors of Notch |
|  | Neighbors of lectin |
|  | Neighbors of fibrinogen |
|  | Neighbors of aldehyde reductase |
|  | Neighbors of interleukin-35 |
|  | Neighbors of TSH |
|  | Neighbors of Ang II receptor |
|  | Neighbors of superoxide dismutase |
|  | Neighbors of proteasome endopeptidase complex |
|  | Neighbors of class nuclear receptor with C4 zinc fingers |
|  | Neighbors of non-coding RNA |
|  | Neighbors of STAT5 |
|  | Neighbors of vitronectin receptor (integrin) |
|  | Neighbors of WNT |
|  | Neighbors of gonadotropins |
|  | Neighbors of PP2B |
|  | Neighbors of phospholipase |
|  | Neighbors of AHR subfamily |
|  | Neighbors of ITG |
|  | Neighbors of endostatins |
|  | Neighbors of CD3 complex |
|  | Neighbors of relaxin |
|  | Neighbors of angiotensin receptor |
|  | Neighbors of RA receptor |
|  | Neighbors of histone |
|  | Neighbors of Rho kinase |
|  | Neighbors of PDGF receptor |
|  | Neighbors of neuropeptides |
|  | Neighbors of pancreatic elastase |
|  | Neighbors of adrenoceptor |
|  | Neighbors of TLR4/Myd88 |
|  | Neighbors of TNF-alpha receptor |
|  | comon ps\_pos, pprarg\_neg targets |
|  | Model of PPARG signaling in psoriasis |
|  | PPARG negative regulators and targets |
|  | Model of PPARG related pathways in psoriasis (short version) |
|  | sars-cov no binding |
|  | Overview. Diseases of the skin (chapter 11) |
|  | Overview. Diseases of the respiratory (chapter 9) |
|  | Overview. Diseases of the genitourinary system (chapter 13) |
|  | 1\_Differentiation of psoriatic T cells |
|  | 3\_TGFB signaling provokes endochondral ossification with osteophyte formation |
|  | 3\_The role of interstitial fibroblasts in pyelonephritis |
|  | 3\_Mesangial cell dysfunction in glomerulonephritis |
|  | 2\_Podocyte dysfunction in glomerulonephritis |
|  | 2\_MYOC-associated glaucoma |
|  | 3\_Increased proliferation of pulmonary artery smooth muscle cells (PASMCs) in familial forms of pulmonary hypertension |
|  | 2\_Cardiomyocyte hypertrophy |
|  | 1\_Cigarette smoke causes dysfunction and production of inflammatory mediators in alveolar epithelial cells |
|  | 3\_Role of alveolar macrophages in pulmonary tissue destruction in COPD |
|  | 2\_2\_Eosinophilia and neutrophilia in asthma: eosinophil apoptosis |
|  | 4\_2\_Airway smooth muscle cells proliferation and airway tissue remodeling |
|  | 4\_1\_Polymorphisms associated with inflammatory bowel diseases |
|  | 1\_2\_Feedback relations between coagulation and fibrinolysis |
|  | 1\_SERPINA1-associated pulmonary emphysema |
|  | 1\_Antigen-presenting cells promote islet dysfunction and immune system activation in T1D |
|  | 2\_Defective tolerance of autoreactive T-cell in T1D |
|  | 1\_Microangiopathy in diabetic neuropathy |
|  | drug-target moltransport |
|  | drug-target molsynthesis |
|  | Differentiation of psoriatic T cells |

---

|  |  |
| --- | --- |
| Group | Genes with Mutations Associated with Atherosclerosis |
|  | Genes Associated with Systemic Lupus Erythematosus |
|  | Genes with Mutations Associated with Obesity |
|  | Genes with Mutations Associated with Cystic Fibrosis |
|  | Genes with Mutations Associated with Systemic Scleroderma |
|  | Genes with Polymorphisms Associated with Migraine |
|  | Genes with Mutations Associated with Endometriosis |
|  | Chemokines, Growth Factors and Receptors Involved in Cancer Cells Chemotaxis |
|  | other partners for SARS |

---

|  |  |
| --- | --- |
| Source | Pathway Studio Ontology |
|  | curated |
|  | GO component |
|  | ResNet |

---

|  |  |
| --- | --- |
| MedScan ID | 7040 |

---

|  |  |
| --- | --- |
| LocusLink ID | 7040 |
|  | 21803 |
|  | 59086 |
|  | 43846 |
|  | 282089 |
|  | 359834 |
|  | 337700 |

---

|  |  |
| --- | --- |
| Alias | TGF eta |
|  | TGF-beta 1 |
|  | TGF1\_RAT |
|  | tumor growth factor beta |
|  | tumor growth factor beta 1 |
|  | transforming growth factor beta 1 |
|  | TGF-beta I |
|  | trans-forming growth factor beta |
|  | transforming growth b1 |
|  | TGF1\_HUMAN |
|  | TGFB1 |
|  | IBDIMDE |
|  | tumor growth factor-b |
|  | Tgfb I |
|  | trans-forming growth factor beta 1 |
|  | TGFB |
|  | Camurati-Engelmann disease protein |
|  | tumor growth factor b-1 |
|  | TGF beta |
|  | transforming growth factor (TGF)-beta |
|  | TGF-B1 |
|  | beta 1 transforming growth factor |
|  | Camurati-Engelmann disease gene |
|  | prepro-transforming growth factor beta-1 |
|  | transforming growth factor beta |
|  | CED |
|  | LAP |
|  | DPD1 |
|  | TGFbeta |
|  | TGF-beta-1 |
|  | latency-associated peptide |
|  | transforming growth factor beta-1 proprotein |
|  | Tgfb-1 |
|  | TGFbeta1 |
|  | TGF-beta1 |
|  | transforming growth factor beta-1 |
|  | tumour growth factor beta |
|  | transforming growth factor, beta-1 |
|  | regulatory protein |
|  | transforming growth factor-beta 1 |
|  | TGF-beta 1 protein |
|  | transforming growth factor, beta |
|  | diaphyseal dysplasia 1, progressive |
|  | DPD I |
|  | Transforming growth factor beta 1 precursor |
|  | transforming growth factor, beta 1 |
|  | transforming growth factor, beta 1 (Camurati-Engelmann disease) |
|  | diaphyseal dysplasia 1, progressive (Camurati-Engelmann disease) |
|  | Camurati-Engelmann disease |
|  | diaphyseal dysplasia 1 |
|  | diaphyseal dysplasia I |
|  | beta 1 (Camurati-Engelmann disease) |
|  | transforming growth factor beta-1 precursor |
|  | TGF1 |
|  | HGNC:2997 |
|  | wu:fb13a07 |
|  | xx:ai39657 |
|  | ai39657 |
|  | beta 1 |
|  | chromosome |
|  | scaffold |
|  | transforming growth factor |

---

|  |  |
| --- | --- |
| GO ID | 0003823 |
|  | 0005125 |
|  | 0019899 |
|  | 0008083 |
|  | 0047485 |
|  | 0046982 |
|  | 0042803 |
|  | 0043539 |
|  | 0005160 |
|  | 0034713 |
|  | 0005114 |
|  | 0034714 |
|  | 0006754 |
|  | 0000165 |
|  | 0007219 |
|  | 0007183 |
|  | 0060395 |
|  | 0043029 |
|  | 0002460 |
|  | 0007568 |
|  | 0003180 |
|  | 0060751 |
|  | 0007050 |
|  | 0048468 |
|  | 0016477 |
|  | 0045216 |
|  | 0006874 |
|  | 0071549 |
|  | 1990314 |
|  | 0071479 |
|  | 0071260 |
|  | 0071407 |
|  | 0071560 |
|  | 0002062 |
|  | 0007182 |
|  | 0002248 |
|  | 0019221 |
|  | 0009817 |
|  | 0048565 |
|  | 1990402 |
|  | 0007492 |
|  | 0007173 |
|  | 0001837 |
|  | 0019049 |
|  | 0085029 |
|  | 0097191 |
|  | 0060325 |
|  | 0007565 |
|  | 0060364 |
|  | 0008354 |
|  | 0007507 |
|  | 0003179 |
|  | 0002244 |
|  | 0030214 |
|  | 0006954 |
|  | 0048839 |
|  | 0070306 |
|  | 0050900 |
|  | 0031663 |
|  | 0097421 |
|  | 0048535 |
|  | 0010742 |
|  | 0060744 |
|  | 0031293 |
|  | 0007093 |
|  | 0032943 |
|  | 0046716 |
|  | 0042552 |
|  | 0043011 |
|  | 0042130 |
|  | 0070168 |
|  | 0043537 |
|  | 0045786 |
|  | 0045596 |
|  | 0030308 |
|  | 0008285 |
|  | 0022408 |
|  | 0045918 |
|  | 0050680 |
|  | 0010716 |
|  | 0045599 |
|  | 0010629 |
|  | 0060965 |
|  | 1900126 |
|  | 0032700 |
|  | 0010936 |
|  | 0045930 |
|  | 0045662 |
|  | 0007406 |
|  | 0030279 |
|  | 0050765 |
|  | 1903799 |
|  | 1903077 |
|  | 0001933 |
|  | 0051280 |
|  | 0048642 |
|  | 0000122 |
|  | 0045892 |
|  | 0030512 |
|  | 0001843 |
|  | 0021915 |
|  | 0014003 |
|  | 0043932 |
|  | 0030316 |
|  | 0060389 |
|  | 0006796 |
|  | 0002576 |
|  | 0070374 |
|  | 0043406 |
|  | 1901666 |
|  | 0051092 |
|  | 0060391 |
|  | 0043065 |
|  | 0043536 |
|  | 0030501 |
|  | 0090190 |
|  | 2000727 |
|  | 0071158 |
|  | 0051781 |
|  | 0030335 |
|  | 0008284 |
|  | 0032270 |
|  | 0050921 |
|  | 0032967 |
|  | 0050679 |
|  | 0010718 |
|  | 0031536 |
|  | 1901203 |
|  | 0010763 |
|  | 0048146 |
|  | 0010628 |
|  | 0035066 |
|  | 0031065 |
|  | 0032740 |
|  | 0048298 |
|  | 0014008 |
|  | 0071677 |
|  | 0042482 |
|  | 0010862 |
|  | 0033138 |
|  | 0010800 |
|  | 0050731 |
|  | 0043552 |
|  | 1902895 |
|  | 1903800 |
|  | 0031334 |
|  | 0035307 |
|  | 0042307 |
|  | 0051897 |
|  | 1900182 |
|  | 0001934 |
|  | 0050714 |
|  | 1903911 |
|  | 0045591 |
|  | 0051152 |
|  | 0032930 |
|  | 0045944 |
|  | 2000679 |
|  | 0045893 |
|  | 0010575 |
|  | 0043117 |
|  | 0006611 |
|  | 0043491 |
|  | 0006468 |
|  | 0032801 |
|  | 0051101 |
|  | 0043408 |
|  | 0060390 |
|  | 2000249 |
|  | 0042981 |
|  | 0051098 |
|  | 0060312 |
|  | 0060762 |
|  | 0061035 |
|  | 0030334 |
|  | 0042127 |
|  | 1905005 |
|  | 0032667 |
|  | 1902893 |
|  | 0042306 |
|  | 0002028 |
|  | 0016202 |
|  | 0017015 |
|  | 0045066 |
|  | 0070723 |
|  | 0032355 |
|  | 0009749 |
|  | 0001666 |
|  | 0035902 |
|  | 0034616 |
|  | 0032570 |
|  | 1902074 |
|  | 0033280 |
|  | 0009611 |
|  | 0007435 |
|  | 0002513 |
|  | 0007179 |
|  | 1905313 |
|  | 0001657 |
|  | 0001570 |
|  | 0055010 |
|  | 0005796 |
|  | 0030424 |
|  | 0072562 |
|  | 0009986 |
|  | 0062023 |
|  | 0005737 |
|  | 0031012 |
|  | 0005576 |
|  | 0005615 |
|  | 0005902 |
|  | 0043025 |
|  | 0005634 |
|  | 0005886 |
|  | 0031093 |
|  | 0002362 |
|  | 0042110 |
|  | 0030217 |
|  | 0072540 |
|  | 0009887 |
|  | 0031100 |
|  | 0001775 |
|  | 0008283 |
|  | 0071363 |
|  | 0061448 |
|  | 0006952 |
|  | 0030879 |
|  | 0001763 |
|  | 0050868 |
|  | 0050777 |
|  | 0032829 |
|  | 0010468 |
|  | 0045589 |
|  | 0010033 |
|  | 0007519 |
|  | 0001501 |
|  | 0042060 |
|  | 0030141 |
|  | 0030324 |
|  | 0001558 |
|  | 0010469 |
|  | 0042493 |
|  | 0014070 |
|  | 0009314 |
|  | 0000060 |
|  | 0001948 |
|  | 0007184 |
|  | 0046732 |
|  | 0016049 |
|  | 0008156 |
|  | 0005578 |
|  | 0040007 |
|  | 0007596 |
|  | 0030198 |
|  | 0019048 |
|  | 0030168 |
|  | 2000628 |
|  | 0019058 |
|  | 0005623 |
|  | 0009790 |
|  | 0008219 |
|  | 0043499 |
|  | 0006917 |
|  | 0031575 |
|  | 0016563 |
|  | 0043193 |
|  | 0010552 |
|  | 0051789 |
|  | 0071445 |
|  | 0016481 |
|  | 0042476 |
|  | 0005515 |
|  | 0045595 |
|  | 0006916 |
|  | 0000074 |
|  | 0007267 |
|  | 0046888 |
|  | 0003677 |
|  | 0006306 |
|  | 0008170 |
|  | 0007515 |
|  | 0008220 |
|  | 0007275 |
|  | 0045941 |

---

|  |  |
| --- | --- |
| KEGG ID | hsa:7040 |
|  | mmu:21803 |
|  | rno:59086 |

---

|  |  |
| --- | --- |
| MedLine Reference | 10504497 |
|  | 10967130 |
|  | 11145590 |
|  | 11597910 |
|  | 11769973 |
|  | 11784717 |
|  | 11842006 |
|  | 11872640 |
|  | 11904165 |
|  | 11906188 |
|  | 11906205 |
|  | 11961311 |
|  | 12002441 |
|  | 12021073 |
|  | 12060054 |
|  | 12130550 |
|  | 12150952 |
|  | 12199527 |
|  | 12208371 |
|  | 12209600 |
|  | 12225958 |
|  | 12239085 |
|  | 12372783 |
|  | 12410804 |
|  | 12466133 |
|  | 12477932 |
|  | 12484516 |
|  | 12496370 |
|  | 12538588 |
|  | 12545247 |
|  | 12598227 |
|  | 12645077 |
|  | 12646048 |
|  | 12697724 |
|  | 12702545 |
|  | 12704789 |
|  | 12712323 |
|  | 12730080 |
|  | 12794007 |
|  | 12806594 |
|  | 12815054 |
|  | 12837295 |
|  | 12865256 |
|  | 12867422 |
|  | 12876066 |
|  | 12876289 |
|  | 12937160 |
|  | 12970361 |
|  | 12971963 |
|  | 14514686 |
|  | 14575307 |
|  | 14575314 |
|  | 14597484 |
|  | 14612425 |
|  | 14623275 |
|  | 14623893 |
|  | 14633629 |
|  | 14648590 |
|  | 14670985 |
|  | 14697288 |
|  | 14704634 |
|  | 14708940 |
|  | 14717916 |
|  | 14726452 |
|  | 14960316 |
|  | 14967845 |
|  | 15019289 |
|  | 15047629 |
|  | 15153551 |
|  | 15184388 |
|  | 15187003 |
|  | 15192047 |
|  | 15201552 |
|  | 15222754 |
|  | 15251335 |
|  | 15253713 |
|  | 15316360 |
|  | 15334678 |
|  | 15450206 |
|  | 15492480 |
|  | 15550525 |
|  | 15604136 |
|  | 15644487 |
|  | 15652405 |
|  | 15677311 |
|  | 15677465 |
|  | 15746252 |
|  | 15781474 |
|  | 15808558 |
|  | 15855808 |
|  | 15964416 |
|  | 15994428 |
|  | 16005428 |
|  | 16019429 |
|  | 16020542 |
|  | 16097048 |
|  | 16103255 |
|  | 16105028 |
|  | 16146787 |
|  | 16157589 |
|  | 16177030 |
|  | 16182431 |
|  | 16183734 |
|  | 16246848 |
|  | 16272332 |
|  | 16362724 |
|  | 16371439 |
|  | 16380081 |
|  | 16449286 |
|  | 16473861 |
|  | 16546826 |
|  | 16549155 |
|  | 16549518 |
|  | 16685417 |
|  | 16690877 |
|  | 16770245 |
|  | 16778334 |
|  | 16778384 |
|  | 16785237 |
|  | 16935907 |
|  | 2349108 |
|  | 7852342 |
|  | 8106553 |
|  | 9757003 |
|  | 9779826 |
|  | 10021345 |
|  | 10027923 |
|  | 10030593 |
|  | 10084677 |
|  | 10092113 |
|  | 10191100 |
|  | 10215326 |
|  | 10224044 |
|  | 10341708 |
|  | 10349630 |
|  | 10349636 |
|  | 10411901 |
|  | 10416598 |
|  | 10433821 |
|  | 10438931 |
|  | 10446271 |
|  | 10450746 |
|  | 10477295 |
|  | 10491004 |
|  | 10491283 |
|  | 10521498 |
|  | 10521784 |
|  | 10534616 |
|  | 10562293 |
|  | 10602018 |
|  | 10611318 |
|  | 10622417 |
|  | 10623661 |
|  | 10720419 |
|  | 10722796 |
|  | 10725853 |
|  | 10741419 |
|  | 10744631 |
|  | 10813854 |
|  | 10848613 |
|  | 10886500 |
|  | 10926756 |
|  | 10929204 |
|  | 10945464 |
|  | 10976050 |
|  | 11042159 |
|  | 11074608 |
|  | 11076861 |
|  | 11106407 |
|  | 11123210 |
|  | 11124112 |
|  | 11154238 |
|  | 11159184 |
|  | 11168809 |
|  | 11217851 |
|  | 11280770 |
|  | 11285272 |
|  | 11304693 |
|  | 11329064 |
|  | 11337363 |
|  | 11342667 |
|  | 11402321 |
|  | 11458389 |
|  | 11481479 |
|  | 11509733 |
|  | 11514601 |
|  | 11518504 |
|  | 11536370 |
|  | 11560950 |
|  | 11599050 |
|  | 11606377 |
|  | 11641043 |
|  | 11675471 |
|  | 11677049 |
|  | 11684435 |
|  | 11711431 |
|  | 11722849 |
|  | 11781182 |
|  | 11784073 |
|  | 11799106 |
|  | 11826761 |
|  | 11830573 |
|  | 11850179 |
|  | 11856815 |
|  | 11857453 |
|  | 11901187 |
|  | 11981827 |
|  | 12020424 |
|  | 12027416 |
|  | 12027867 |
|  | 12057913 |
|  | 12069494 |
|  | 12090759 |
|  | 12117781 |
|  | 12119348 |
|  | 12130649 |
|  | 12141444 |
|  | 12163410 |
|  | 12185452 |
|  | 12192060 |
|  | 12208849 |
|  | 12211442 |
|  | 12218417 |
|  | 12242711 |
|  | 12374793 |
|  | 12381916 |
|  | 12384514 |
|  | 12393578 |
|  | 12393681 |
|  | 12417574 |
|  | 12421948 |
|  | 12421979 |
|  | 12433697 |
|  | 12466851 |
|  | 12469906 |
|  | 12489180 |
|  | 12511596 |
|  | 12516540 |
|  | 12525489 |
|  | 12531561 |
|  | 12545162 |
|  | 12566226 |
|  | 12588999 |
|  | 12598898 |
|  | 12624490 |
|  | 12626500 |
|  | 12626524 |
|  | 12634787 |
|  | 12637528 |
|  | 12646629 |
|  | 12666199 |
|  | 12666204 |
|  | 12697700 |
|  | 12707256 |
|  | 12707335 |
|  | 12707339 |
|  | 12707360 |
|  | 12720300 |
|  | 12739785 |
|  | 12794116 |
|  | 12804760 |
|  | 12808151 |
|  | 12812985 |
|  | 12815042 |
|  | 12815630 |
|  | 12829028 |
|  | 12837285 |
|  | 12842083 |
|  | 12842983 |
|  | 12869309 |
|  | 12871697 |
|  | 12880367 |
|  | 12888549 |
|  | 12896968 |
|  | 12920022 |
|  | 12933797 |
|  | 12933825 |
|  | 12972711 |
|  | 12975472 |
|  | 13679037 |
|  | 1373953 |
|  | 1374351 |
|  | 1436033 |
|  | 14499634 |
|  | 14507636 |
|  | 14527958 |
|  | 14550911 |
|  | 14607897 |
|  | 14609524 |
|  | 14612410 |
|  | 14617508 |
|  | 14617750 |
|  | 14633599 |
|  | 14635047 |
|  | 14643888 |
|  | 14656760 |
|  | 14657019 |
|  | 14687548 |
|  | 14697224 |
|  | 14697238 |
|  | 14697364 |
|  | 14699019 |
|  | 14704188 |
|  | 14707053 |
|  | 14732719 |
|  | 14733613 |
|  | 14734769 |
|  | 14749725 |
|  | 14755243 |
|  | 14761479 |
|  | 14761480 |
|  | 14764882 |
|  | 14766723 |
|  | 14769378 |
|  | 1497904 |
|  | 14985127 |
|  | 14988498 |
|  | 15004187 |
|  | 15005577 |
|  | 15016653 |
|  | 1502148 |
|  | 15037657 |
|  | 15039225 |
|  | 15059931 |
|  | 15070759 |
|  | 15073150 |
|  | 15084245 |
|  | 15086458 |
|  | 15100250 |
|  | 15116295 |
|  | 15121012 |
|  | 15135063 |
|  | 15136142 |
|  | 15147727 |
|  | 15150273 |
|  | 15153523 |
|  | 15162832 |
|  | 15175265 |
|  | 15195967 |
|  | 15206957 |
|  | 15215176 |
|  | 15215210 |
|  | 15232609 |
|  | 15239668 |
|  | 15240716 |
|  | 15251394 |
|  | 15254903 |
|  | 15260977 |
|  | 15264213 |
|  | 15282343 |
|  | 15305294 |
|  | 15305298 |
|  | 15306544 |
|  | 15317845 |
|  | 15342483 |
|  | 15342643 |
|  | 15356151 |
|  | 15389721 |
|  | 1545145 |
|  | 15464579 |
|  | 15466481 |
|  | 15504975 |
|  | 15514019 |
|  | 15516833 |
|  | 15536133 |
|  | 15546004 |
|  | 15548578 |
|  | 15569352 |
|  | 15572143 |
|  | 15579311 |
|  | 15593298 |
|  | 15599395 |
|  | 15604101 |
|  | 15604265 |
|  | 15607422 |
|  | 15607541 |
|  | 15629141 |
|  | 15649710 |
|  | 15654327 |
|  | 15664007 |
|  | 15665119 |
|  | 15696185 |
|  | 15699137 |
|  | 15708558 |
|  | 15708853 |
|  | 15710627 |
|  | 15721312 |
|  | 15734730 |
|  | 15753207 |
|  | 15758078 |
|  | 15760451 |
|  | 1577201 |
|  | 15782092 |
|  | 15784407 |
|  | 15799023 |
|  | 15809351 |
|  | 15820682 |
|  | 15837559 |
|  | 15843580 |
|  | 15845540 |
|  | 15852008 |
|  | 15855773 |
|  | 15878400 |
|  | 15879087 |
|  | 15879091 |
|  | 15896309 |
|  | 15922720 |
|  | 15923183 |
|  | 15937546 |
|  | 15955531 |
|  | 15963767 |
|  | 15983059 |
|  | 15983197 |
|  | 15993878 |
|  | 1602131 |
|  | 1602133 |
|  | 16025000 |
|  | 16029193 |
|  | 16029195 |
|  | 16039615 |
|  | 16045809 |
|  | 16049327 |
|  | 16054705 |
|  | 16055724 |
|  | 16093352 |
|  | 16115815 |
|  | 16120811 |
|  | 1612587 |
|  | 16141072 |
|  | 16141073 |
|  | 16148944 |
|  | 16164639 |
|  | 16177068 |
|  | 16179614 |
|  | 1618373 |
|  | 16186809 |
|  | 16192636 |
|  | 16207831 |
|  | 16210663 |
|  | 16236725 |
|  | 16260615 |
|  | 16260650 |
|  | 16263412 |
|  | 16286241 |
|  | 16286245 |
|  | 16288910 |
|  | 16294321 |
|  | 16323703 |
|  | 16325506 |
|  | 16327802 |
|  | 16330325 |
|  | 16354837 |
|  | 1637551 |
|  | 1637552 |
|  | 1638085 |
|  | 16384533 |
|  | 16434965 |
|  | 16435884 |
|  | 16470614 |
|  | 16473830 |
|  | 16478921 |
|  | 16484225 |
|  | 16530041 |
|  | 16537572 |
|  | 16543950 |
|  | 16581977 |
|  | 16628253 |
|  | 16631603 |
|  | 16641085 |
|  | 16647563 |
|  | 16648837 |
|  | 16670772 |
|  | 16702209 |
|  | 16702541 |
|  | 16723700 |
|  | 16738314 |
|  | 16766169 |
|  | 1676977 |
|  | 16773716 |
|  | 16788095 |
|  | 16813524 |
|  | 16829211 |
|  | 16839530 |
|  | 16863909 |
|  | 16866871 |
|  | 1687650 |
|  | 16879310 |
|  | 16903915 |
|  | 16918694 |
|  | 16921114 |
|  | 16921522 |
|  | 16942751 |
|  | 1696875 |
|  | 16973386 |
|  | 16990280 |
|  | 16996057 |
|  | 16998492 |
|  | 17015622 |
|  | 17056535 |
|  | 17058229 |
|  | 17071578 |
|  | 17074765 |
|  | 1707784 |
|  | 17098745 |
|  | 17111364 |
|  | 1723948 |
|  | 1726565 |
|  | 1733675 |
|  | 1764993 |
|  | 1811953 |
|  | 1821856 |
|  | 1879607 |
|  | 1923524 |
|  | 1955457 |
|  | 1970330 |
|  | 1970795 |
|  | 1981054 |
|  | 1986255 |
|  | 2015789 |
|  | 2069871 |
|  | 211512 |
|  | 2206556 |
|  | 2209468 |
|  | 2226202 |
|  | 2375595 |
|  | 2401212 |
|  | 2842249 |
|  | 3007454 |
|  | 3226728 |
|  | 3459257 |
|  | 7485382 |
|  | 7485998 |
|  | 7493021 |
|  | 7507352 |
|  | 7514900 |
|  | 7515916 |
|  | 7519644 |
|  | 7520838 |
|  | 7523056 |
|  | 7527545 |
|  | 7541677 |
|  | 7554934 |
|  | 7555731 |
|  | 7574296 |
|  | 7586747 |
|  | 7594497 |
|  | 7600998 |
|  | 7601910 |
|  | 7632846 |
|  | 7635512 |
|  | 7636210 |
|  | 7636223 |
|  | 7654366 |
|  | 7662961 |
|  | 7673336 |
|  | 7673733 |
|  | 7685120 |
|  | 7687212 |
|  | 7708687 |
|  | 7722461 |
|  | 7745433 |
|  | 7760369 |
|  | 7774812 |
|  | 7813789 |
|  | 7819129 |
|  | 7819439 |
|  | 7821214 |
|  | 7826627 |
|  | 7835921 |
|  | 7838530 |
|  | 7842167 |
|  | 7848824 |
|  | 7856732 |
|  | 7856734 |
|  | 7860639 |
|  | 7883078 |
|  | 7883868 |
|  | 7890140 |
|  | 7890758 |
|  | 7925771 |
|  | 7954410 |
|  | 7958907 |
|  | 7962115 |
|  | 7964480 |
|  | 7964554 |
|  | 7980597 |
|  | 8002998 |
|  | 8009224 |
|  | 8020153 |
|  | 8021288 |
|  | 8023963 |
|  | 8050368 |
|  | 8051207 |
|  | 8051399 |
|  | 8078307 |
|  | 8100843 |
|  | 8104872 |
|  | 8106554 |
|  | 8113105 |
|  | 8113392 |
|  | 8119124 |
|  | 8119140 |
|  | 8119154 |
|  | 8119973 |
|  | 8125201 |
|  | 8138038 |
|  | 8138592 |
|  | 8148055 |
|  | 8156105 |
|  | 8164195 |
|  | 8188294 |
|  | 8197206 |
|  | 8234339 |
|  | 8253379 |
|  | 8288260 |
|  | 8299934 |
|  | 8301216 |
|  | 8305211 |
|  | 8317552 |
|  | 8344985 |
|  | 8358713 |
|  | 8358866 |
|  | 8358867 |
|  | 8364887 |
|  | 8390853 |
|  | 8406668 |
|  | 8409446 |
|  | 8421714 |
|  | 8499342 |
|  | 8522200 |
|  | 8528505 |
|  | 8530024 |
|  | 8554587 |
|  | 8603072 |
|  | 8608234 |
|  | 8618872 |
|  | 8621238 |
|  | 8623195 |
|  | 8632016 |
|  | 8642342 |
|  | 8656060 |
|  | 8662221 |
|  | 8667617 |
|  | 8726224 |
|  | 8752208 |
|  | 8760252 |
|  | 8769667 |
|  | 8791525 |
|  | 8791534 |
|  | 8806818 |
|  | 8812484 |
|  | 8858565 |
|  | 8883333 |
|  | 8885230 |
|  | 8889548 |
|  | 8916368 |
|  | 8918254 |
|  | 8948582 |
|  | 8950991 |
|  | 8976197 |
|  | 9008713 |
|  | 9010342 |
|  | 9013979 |
|  | 9014832 |
|  | 9020852 |
|  | 9088981 |
|  | 9097023 |
|  | 9102398 |
|  | 9132278 |
|  | 9164977 |
|  | 9169087 |
|  | 9187100 |
|  | 9203584 |
|  | 9239404 |
|  | 9281382 |
|  | 9344654 |
|  | 9358754 |
|  | 9367982 |
|  | 9388486 |
|  | 9390516 |
|  | 9390635 |
|  | 9393997 |
|  | 9414479 |
|  | 9457904 |
|  | 9525700 |
|  | 9607560 |
|  | 9618146 |
|  | 9647219 |
|  | 9657149 |
|  | 9662371 |
|  | 9701466 |
|  | 9716414 |
|  | 9724050 |
|  | 9730989 |
|  | 9815262 |
|  | 9839358 |
|  | 9858490 |
|  | 9858566 |
|  | 9878080 |
|  | 9882480 |
|  | 9884332 |
|  | 9921650 |
|  | 9927325 |
|  | 9927592 |
|  | 10025398 |
|  | 10085302 |
|  | 10376933 |
|  | 10383378 |
|  | 10431160 |
|  | 10457363 |
|  | 10631145 |
|  | 10652271 |
|  | 10716993 |
|  | 10843814 |
|  | 10973241 |
|  | 11157754 |
|  | 11172812 |
|  | 11374867 |
|  | 11456401 |
|  | 11483955 |
|  | 11502704 |
|  | 11604083 |
|  | 11740340 |
|  | 11750277 |
|  | 11769974 |
|  | 11771728 |
|  | 11776328 |
|  | 11784716 |
|  | 11796824 |
|  | 11803605 |
|  | 11824477 |
|  | 11836565 |
|  | 11839086 |
|  | 11867550 |
|  | 11876742 |
|  | 11876761 |
|  | 11906036 |
|  | 11908736 |
|  | 11911944 |
|  | 11912197 |
|  | 11924651 |
|  | 11925630 |
|  | 11928807 |
|  | 11934870 |
|  | 11970960 |
|  | 11991670 |
|  | 11991675 |
|  | 11998866 |
|  | 12000722 |
|  | 12015308 |
|  | 12020803 |
|  | 12021923 |
|  | 12032592 |
|  | 12041672 |
|  | 12046078 |
|  | 12055267 |
|  | 12057905 |
|  | 12061838 |
|  | 12062452 |
|  | 12068984 |
|  | 12081893 |
|  | 12081894 |
|  | 12082048 |
|  | 12085100 |
|  | 12097320 |
|  | 12099698 |
|  | 12107734 |
|  | 12140283 |
|  | 12150293 |
|  | 12161098 |
|  | 12161428 |
|  | 12163012 |
|  | 12163055 |
|  | 12168782 |
|  | 12170265 |
|  | 12171249 |
|  | 12172787 |
|  | 12174377 |
|  | 12175481 |
|  | 12186868 |
|  | 12189134 |
|  | 12202226 |
|  | 12202478 |
|  | 12209716 |
|  | 12219028 |
|  | 12223346 |
|  | 12234017 |
|  | 12352892 |
|  | 12366695 |
|  | 12370400 |
|  | 12370804 |
|  | 12376363 |
|  | 12379307 |
|  | 12382579 |
|  | 12393416 |
|  | 12393612 |
|  | 12397374 |
|  | 12411321 |
|  | 12413771 |
|  | 12421823 |
|  | 12431651 |
|  | 12431652 |
|  | 12432546 |
|  | 12438348 |
|  | 12441075 |
|  | 12453980 |
|  | 12454225 |
|  | 12457461 |
|  | 12479636 |
|  | 12485831 |
|  | 12485877 |
|  | 12489179 |
|  | 12489185 |
|  | 12493411 |
|  | 12493741 |
|  | 12500218 |
|  | 12514788 |
|  | 12515830 |
|  | 12529270 |
|  | 12531695 |
|  | 12531696 |
|  | 12531888 |
|  | 12540377 |
|  | 12547711 |
|  | 12548199 |
|  | 12559970 |
|  | 12560323 |
|  | 12569017 |
|  | 12579267 |
|  | 12579348 |
|  | 12589095 |
|  | 12595498 |
|  | 12607775 |
|  | 12615364 |
|  | 12637138 |
|  | 12649573 |
|  | 12654639 |
|  | 12667558 |
|  | 12669309 |
|  | 12675860 |
|  | 12682078 |
|  | 12700666 |
|  | 12723985 |
|  | 12743121 |
|  | 12747448 |
|  | 12751024 |
|  | 12753290 |
|  | 12754205 |
|  | 12758167 |
|  | 12772773 |
|  | 12775566 |
|  | 12787424 |
|  | 12794244 |
|  | 12795790 |
|  | 12802498 |
|  | 12810668 |
|  | 1281686 |
|  | 12817760 |
|  | 12821938 |
|  | 12824005 |
|  | 12838101 |
|  | 12843182 |
|  | 12850832 |
|  | 12857600 |
|  | 12858451 |
|  | 12874450 |
|  | 12875973 |
|  | 12879019 |
|  | 12879228 |
|  | 12893825 |
|  | 12894997 |
|  | 12898374 |
|  | 12902338 |
|  | 12911534 |
|  | 12911563 |
|  | 12917425 |
|  | 12938093 |
|  | 12943995 |
|  | 12953061 |
|  | 12960355 |
|  | 13679381 |
|  | 14504277 |
|  | 14504444 |
|  | 14507446 |
|  | 14510802 |
|  | 14514699 |
|  | 14525983 |
|  | 14529885 |
|  | 14531804 |
|  | 14576166 |
|  | 14576954 |
|  | 14583433 |
|  | 14585325 |
|  | 14595120 |
|  | 14600158 |
|  | 14607563 |
|  | 14607699 |
|  | 14612931 |
|  | 14614718 |
|  | 14633126 |
|  | 14634021 |
|  | 14635011 |
|  | 14636845 |
|  | 14637150 |
|  | 14642613 |
|  | 14644155 |
|  | 14667198 |
|  | 14672557 |
|  | 14675189 |
|  | 14676209 |
|  | 14712482 |
|  | 14720431 |
|  | 14722617 |
|  | 14737114 |
|  | 14751563 |
|  | 14755254 |
|  | 14757763 |
|  | 14976204 |
|  | 14988407 |
|  | 14991065 |
|  | 14993265 |
|  | 15000149 |
|  | 15001984 |
|  | 15003992 |
|  | 15009180 |
|  | 15018667 |
|  | 15033015 |
|  | 15033492 |
|  | 15034041 |
|  | 15034927 |
|  | 15044465 |
|  | 15044491 |
|  | 15057277 |
|  | 15066699 |
|  | 15068691 |
|  | 15086490 |
|  | 15100259 |
|  | 15102678 |
|  | 15102771 |
|  | 15113633 |
|  | 15114294 |
|  | 15132952 |
|  | 15133024 |
|  | 15133032 |
|  | 15159307 |
|  | 15166495 |
|  | 15170816 |
|  | 15175276 |
|  | 15178409 |
|  | 15180953 |
|  | 15184872 |
|  | 15187087 |
|  | 15187109 |
|  | 15191521 |
|  | 15192088 |
|  | 15200451 |
|  | 15203191 |
|  | 15212689 |
|  | 15219857 |
|  | 15220111 |
|  | 15221033 |
|  | 15225617 |
|  | 15231647 |
|  | 15240008 |
|  | 15247230 |
|  | 15248227 |
|  | 15269821 |
|  | 15271980 |
|  | 15284289 |
|  | 15294900 |
|  | 15297631 |
|  | 15308875 |
|  | 15316224 |
|  | 15334054 |
|  | 15337504 |
|  | 15353478 |
|  | 15354859 |
|  | 15359164 |
|  | 15361357 |
|  | 15365652 |
|  | 15368517 |
|  | 15372625 |
|  | 15373772 |
|  | 15377500 |
|  | 15387373 |
|  | 15450129 |
|  | 15467747 |
|  | 15488704 |
|  | 15494412 |
|  | 15498571 |
|  | 15498766 |
|  | 1550960 |
|  | 15522940 |
|  | 15536170 |
|  | 15539010 |
|  | 15546338 |
|  | 15569360 |
|  | 15574511 |
|  | 15593052 |
|  | 15593053 |
|  | 15596839 |
|  | 15598443 |
|  | 15599946 |
|  | 15604209 |
|  | 15609398 |
|  | 15613484 |
|  | 15623538 |
|  | 15632190 |
|  | 15637079 |
|  | 15641096 |
|  | 15654963 |
|  | 15661045 |
|  | 15665522 |
|  | 15671065 |
|  | 15702480 |
|  | 15703175 |
|  | 15705190 |
|  | 15716278 |
|  | 15730046 |
|  | 15732863 |
|  | 15735739 |
|  | 15738684 |
|  | 15754021 |
|  | 15763337 |
|  | 15767424 |
|  | 15770661 |
|  | 15777850 |
|  | 15778389 |
|  | 15778431 |
|  | 15788474 |
|  | 15794197 |
|  | 15799969 |
|  | 15800982 |
|  | 15802940 |
|  | 15803361 |
|  | 15811853 |
|  | 15818397 |
|  | 15843517 |
|  | 15848527 |
|  | 15854667 |
|  | 15856020 |
|  | 15860935 |
|  | 15880049 |
|  | 15881652 |
|  | 15895394 |
|  | 15905358 |
|  | 15913806 |
|  | 15913903 |
|  | 15917723 |
|  | 15927846 |
|  | 15935652 |
|  | 15935832 |
|  | 15941661 |
|  | 15942255 |
|  | 15945506 |
|  | 15946939 |
|  | 15951295 |
|  | 15951665 |
|  | 15955247 |
|  | 15957132 |
|  | 15982639 |
|  | 16003239 |
|  | 16014625 |
|  | 16044414 |
|  | 16046050 |
|  | 16046504 |
|  | 16055706 |
|  | 16082716 |
|  | 16086840 |
|  | 16095497 |
|  | 16098131 |
|  | 16100012 |
|  | 16111879 |
|  | 16113310 |
|  | 16135802 |
|  | 16135988 |
|  | 16144297 |
|  | 16146757 |
|  | 16158060 |
|  | 16179583 |
|  | 16179826 |
|  | 16184401 |
|  | 16187293 |
|  | 16187311 |
|  | 16207846 |
|  | 16209640 |
|  | 16210002 |
|  | 16219695 |
|  | 16222707 |
|  | 16223572 |
|  | 16223775 |
|  | 16245329 |
|  | 16251192 |
|  | 16253118 |
|  | 16253647 |
|  | 16258727 |
|  | 16258965 |
|  | 16259606 |
|  | 16270194 |
|  | 16277007 |
|  | 16285938 |
|  | 16292494 |
|  | 16301119 |
|  | 16307809 |
|  | 16324212 |
|  | 16330131 |
|  | 16336267 |
|  | 16343799 |
|  | 16367920 |
|  | 16378061 |
|  | 16378062 |
|  | 16387180 |
|  | 16400883 |
|  | 16403803 |
|  | 16407286 |
|  | 16412560 |
|  | 16413503 |
|  | 16414983 |
|  | 16423881 |
|  | 16428771 |
|  | 16467160 |
|  | 16482098 |
|  | 16488890 |
|  | 16495290 |
|  | 16496359 |
|  | 16499994 |
|  | 16504679 |
|  | 16521228 |
|  | 16541418 |
|  | 16543220 |
|  | 16552334 |
|  | 16563223 |
|  | 16563228 |
|  | 16571622 |
|  | 16617095 |
|  | 16619041 |
|  | 16621788 |
|  | 16622033 |
|  | 16637060 |
|  | 16646981 |
|  | 16675485 |
|  | 16675963 |
|  | 16677601 |
|  | 16685022 |
|  | 16690986 |
|  | 16698802 |
|  | 16704689 |
|  | 16718633 |
|  | 16720864 |
|  | 16730947 |
|  | 16732186 |
|  | 16733295 |
|  | 16754688 |
|  | 16792132 |
|  | 16799085 |
|  | 16807529 |
|  | 16815840 |
|  | 16820791 |
|  | 16820871 |
|  | 16824504 |
|  | 16828470 |
|  | 16835828 |
|  | 16837650 |
|  | 16843825 |
|  | 16849317 |
|  | 16873204 |
|  | 16877343 |
|  | 16920945 |
|  | 16921482 |
|  | 16933058 |
|  | 16934609 |
|  | 16962935 |
|  | 16969385 |
|  | 16980081 |
|  | 16987065 |
|  | 16987066 |
|  | 16990569 |
|  | 16998703 |
|  | 17003485 |
|  | 17044077 |
|  | 17052957 |
|  | 17067743 |
|  | 17072955 |
|  | 17074098 |
|  | 17080483 |
|  | 17088559 |
|  | 17097497 |
|  | 17100549 |
|  | 17114344 |
|  | 3470709 |
|  | 3487831 |
|  | 3861940 |
|  | 7638106 |
|  | 7686157 |
|  | 7695626 |
|  | 7798269 |
|  | 7803268 |
|  | 7935686 |
|  | 8093006 |
|  | 8235612 |
|  | 8264131 |
|  | 8294518 |
|  | 8336945 |
|  | 8568261 |
|  | 8599089 |
|  | 8617200 |
|  | 9383706 |
|  | 9434787 |
|  | 9521216 |
|  | 9660815 |
|  | 9670843 |
|  | 9813058 |
|  | 9918798 |
|  | 9987082 |
|  | 11062463 |
|  | 11746498 |
|  | 12202987 |
|  | 14679171 |
|  | 15489334 |
|  | 16133992 |
|  | 16263699 |
|  | 16335952 |
|  | 16369764 |
|  | 16423922 |
|  | 16564125 |
|  | 16712948 |
|  | 16754747 |
|  | 16828225 |
|  | 16896935 |
|  | 16904244 |
|  | 16971953 |
|  | 17014924 |
|  | 17023741 |
|  | 17032917 |
|  | 17035229 |
|  | 17076676 |
|  | 17137775 |
|  | 17139467 |
|  | 17145161 |
|  | 17146441 |
|  | 17151785 |
|  | 17164348 |
|  | 17187359 |
|  | 17194537 |
|  | 17198270 |
|  | 17203972 |
|  | 17205957 |
|  | 17209781 |
|  | 17217620 |
|  | 17230494 |
|  | 17234915 |
|  | 17242066 |
|  | 17261758 |
|  | 17268554 |
|  | 17277740 |
|  | 17285762 |
|  | 17288590 |
|  | 17290010 |
|  | 17292826 |
|  | 17305717 |
|  | 17312920 |
|  | 17314099 |
|  | 17319955 |
|  | 17325209 |
|  | 17327236 |
|  | 17332318 |
|  | 17338374 |
|  | 17341608 |
|  | 17341610 |
|  | 17341683 |
|  | 17346099 |
|  | 17347580 |
|  | 17359969 |
|  | 17360530 |
|  | 17360654 |
|  | 17365852 |
|  | 17367219 |
|  | 17368597 |
|  | 17369289 |
|  | 17371954 |
|  | 17376051 |
|  | 17376924 |
|  | 17381064 |
|  | 17382930 |
|  | 17392271 |
|  | 17401617 |
|  | 17404043 |
|  | 17405902 |
|  | 17409139 |
|  | 17415413 |
|  | 17417945 |
|  | 17418380 |
|  | 17418594 |
|  | 17424886 |
|  | 17433803 |
|  | 17438144 |
|  | 17459720 |
|  | 17468136 |
|  | 17469184 |
|  | 17483238 |
|  | 17485238 |
|  | 17486072 |
|  | 17522998 |
|  | 17530715 |
|  | 17540773 |
|  | 17557799 |
|  | 17560118 |
|  | 17570736 |
|  | 17574548 |
|  | 17579118 |
|  | 17591977 |
|  | 17596875 |
|  | 17597658 |
|  | 17598964 |
|  | 17601559 |
|  | 17605296 |
|  | 17620406 |
|  | 17625450 |
|  | 17626784 |
|  | 17638910 |
|  | 17641021 |
|  | 17644809 |
|  | 17700165 |
|  | 17766609 |
|  | 17768377 |
|  | 2982829 |
|  | 3162913 |
|  | 8424942 |
|  | 8424943 |
|  | 8471846 |
|  | 8679613 |
|  | 9150447 |
|  | 9783545 |
|  | 9895299 |
|  | 10852815 |
|  | 11886525 |
|  | 16751781 |
|  | 16865088 |
|  | 16909112 |
|  | 16943282 |
|  | 16978288 |
|  | 17050629 |
|  | 17053163 |
|  | 17126294 |
|  | 17159989 |
|  | 17202367 |
|  | 17204263 |
|  | 17215516 |
|  | 17222412 |
|  | 17237391 |
|  | 17273784 |
|  | 17293478 |
|  | 17318077 |
|  | 17324777 |
|  | 17353357 |
|  | 17360662 |
|  | 17389738 |
|  | 17391668 |
|  | 17391979 |
|  | 17418116 |
|  | 17474154 |
|  | 17478422 |
|  | 17478551 |
|  | 17481928 |
|  | 17485468 |
|  | 17516543 |
|  | 17530708 |
|  | 17543885 |
|  | 17549683 |
|  | 17560204 |
|  | 17560553 |
|  | 17576802 |
|  | 17579023 |
|  | 17579048 |
|  | 17612560 |
|  | 17620361 |
|  | 17634193 |
|  | 17762268 |
|  | 12223100 |
|  | 15214860 |
|  | 16458528 |
|  | 16837651 |
|  | 17068209 |
|  | 17133352 |
|  | 17158884 |
|  | 17203207 |
|  | 17213961 |
|  | 17335076 |
|  | 17352758 |
|  | 17374997 |
|  | 17379848 |
|  | 17392319 |
|  | 17396111 |
|  | 17485851 |
|  | 17530031 |
|  | 17541159 |
|  | 2070682 |
|  | 12441094 |
|  | 12591230 |
|  | 14502602 |
|  | 15456771 |
|  | 15747730 |
|  | 16004987 |
|  | 16390551 |
|  | 1733936 |
|  | 17388018 |
|  | 3153459 |
|  | 15866162 |
|  | 16197550 |
|  | 17003102 |

---

|  |  |
| --- | --- |
| Organism | Homo sapiens {Organism urn:agi-taxid:9606} |
|  | Mus musculus {Organism urn:agi-taxid:10090} |
|  | Rattus norvegicus {Organism urn:agi-taxid:10116} |
|  | Homo sapiens |
|  | Mus musculus |
|  | Rattus norvegicus |
|  | Bos taurus |
|  | Danio rerio |
|  | Human |

---

|  |  |
| --- | --- |
| Mouse chromosome position | 7 13.98 cM |
|  | 7 6.5 cM |

---

|  |  |
| --- | --- |
| OMIM ID | 190180 |
|  | 219700 |
|  | 131300 |
|  | 618213 |
|  | OMIM:190180 |
|  | OMIM:131300 |

---

|  |  |
| --- | --- |
| Rat chromosome position | 1q21 |
|  | 1q22 |

---

|  |  |
| --- | --- |
| Hugo ID | 11766 |
|  | HGNC:11766 |

---

|  |  |
| --- | --- |
| Human chromosome position | 19q13.2 |
|  | 19q13.1 |

---

|  |  |
| --- | --- |
| Swiss-Prot Accession | P01137 |
|  | P01137.2 |
|  | P04202 |
|  | Q3UNK5 |
|  | P04202.1 |
|  | P17246 |
|  | P17246.1 |
|  | A8K792 |
|  | Q9UCG4 |
|  | Q53YM8 |
|  | Q49IK8 |
|  | Q49IL8 |
|  | Q5PY19 |
|  | Q671U8 |
|  | Q671V1 |
|  | Q7Z487 |
|  | Q7Z488 |
|  | Q86YU2 |
|  | Q9R231 |
|  | TGFB1\_HUMAN |
|  | TGFB1\_MOUSE |
|  | TGFB1\_RAT |
|  | Q3UNK5\_MOUSE |

---

|  |  |
| --- | --- |
| PIR ID | A27513 |
|  | A01396 |
|  | S10219 |

---

|  |  |
| --- | --- |
| GenBank ID | NC\_000019 |
|  | NM\_000660 |
|  | NP\_000651 |
|  | XM\_011527242 |
|  | XP\_011525544 |
|  | NG\_013364 |
|  | AC011462 |
|  | AH013154 |
|  | AAQ18641 |
|  | AY059373 |
|  | AAL27646 |
|  | AY576687 |
|  | AAT77143 |
|  | AY576688 |
|  | AAT77144 |
|  | AY871232 |
|  | AAX59016 |
|  | AY871233 |
|  | AAX59017 |
|  | AY871234 |
|  | AAX59018 |
|  | AY871235 |
|  | AAX59019 |
|  | AY871236 |
|  | AAX59020 |
|  | AY871237 |
|  | AAX59021 |
|  | AY871238 |
|  | AAX59022 |
|  | AY871239 |
|  | AAX59023 |
|  | AY871240 |
|  | AAX59024 |
|  | AY871241 |
|  | AAX59025 |
|  | AY871242 |
|  | AAX59026 |
|  | AY871243 |
|  | AAX59027 |
|  | AY871244 |
|  | AAX59028 |
|  | AY871245 |
|  | AAX59029 |
|  | CH471126 |
|  | EAW57032 |
|  | EAW57033 |
|  | CQ759646 |
|  | CAF32686 |
|  | CS674262 |
|  | CAP12291 |
|  | CS805735 |
|  | CAP40172 |
|  | EU338452 |
|  | ACA64647 |
|  | EU338453 |
|  | ACA64648 |
|  | EU338454 |
|  | ACA64649 |
|  | HC919732 |
|  | CBN73463 |
|  | HC919943 |
|  | CBN73468 |
|  | J04431 |
|  | AAN86616 |
|  | JA104601 |
|  | CCA63970 |
|  | LM651041 |
|  | CDW91956 |
|  | LM651042 |
|  | CDW91957 |
|  | LM651043 |
|  | CDW91958 |
|  | LM651044 |
|  | CDW91959 |
|  | LM651045 |
|  | CDW91960 |
|  | LM651046 |
|  | CDW91961 |
|  | LM651047 |
|  | CDW91962 |
|  | LM651048 |
|  | CDW91963 |
|  | LM651049 |
|  | CDW91964 |
|  | LM651050 |
|  | CDW91965 |
|  | LM651051 |
|  | CDW91966 |
|  | LM651052 |
|  | CDW91967 |
|  | LM651053 |
|  | CDW91968 |
|  | LM651054 |
|  | CDW91969 |
|  | LM651055 |
|  | CDW91970 |
|  | LM651056 |
|  | CDW91971 |
|  | LM651057 |
|  | CDW91972 |
|  | LM651058 |
|  | CDW91973 |
|  | LM651059 |
|  | CDW91974 |
|  | X05839 |
|  | CAA29283 |
|  | AI304490 |
|  | AK291907 |
|  | BAF84596 |
|  | AK307742 |
|  | AY820829 |
|  | AAV71148 |
|  | BC000125 |
|  | AAH00125 |
|  | BC001180 |
|  | AAH01180 |
|  | BC022242 |
|  | AAH22242 |
|  | BG115704 |
|  | BM692012 |
|  | BP267938 |
|  | BT007245 |
|  | AAP35909 |
|  | DA935180 |
|  | M38449 |
|  | AAA36735 |
|  | X02812 |
|  | CAA26580 |
|  | P01137 |
|  | NC\_000073 |
|  | NM\_011577 |
|  | NP\_035707 |
|  | AC162614 |
|  | AH003562 |
|  | AAB00138 |
|  | AY340221 |
|  | AAR06153 |
|  | AY342403 |
|  | AAQ75558 |
|  | CH466593 |
|  | EDL24239 |
|  | M57902 |
|  | AAO87818 |
|  | AA510019 |
|  | AA821506 |
|  | AJ009862 |
|  | CAA08900 |
|  | AK144163 |
|  | BAE25742 |
|  | BC013738 |
|  | AAH13738 |
|  | BQ030974 |
|  | CK625059 |
|  | M13177 |
|  | AAA40423 |
|  | P04202 |
|  | NC\_005100 |
|  | NM\_021578 |
|  | NP\_067589 |
|  | AC\_000069 |
|  | AABR07002708 |
|  | AAHX01004316 |
|  | AF105069 |
|  | AAD20222 |
|  | CH473979 |
|  | EDM07998 |
|  | AY550025 |
|  | AAS55640 |
|  | BC076380 |
|  | AAH76380 |
|  | X52498 |
|  | CAA36741 |
|  | P17246 |
|  | NC\_018930 |
|  | AMYH02036132 |
|  | AMYH02036133 |
|  | AY330201 |
|  | AY330202 |
|  | AAQ18642 |
|  | EU832132 |
|  | ACE87482 |
|  | EU832225 |
|  | ACE86795 |
|  | AC\_000029 |
|  | AAHY01060005 |
|  | L42456 |
|  | L42457 |
|  | L42458 |
|  | L42459 |
|  | L42460 |
|  | L42461 |
|  | L42462 |
|  | AC\_000151 |
|  | ABBA01040804 |
|  | ABBA01040805 |
|  | XM\_005259187 |
|  | XP\_005259244 |
|  | AABR06003977 |
|  | NT\_011109 |
|  | NW\_927217 |
|  | NW\_001838496 |
|  | NG\_013091 |
|  | AC\_000062 |
|  | CR592182 |
|  | CR601792 |
|  | CR612251 |
|  | Q49IK8 |
|  | Q49IL8 |
|  | Q5PY19 |
|  | Q671U8 |
|  | Q671V1 |
|  | Q7Z487 |
|  | Q7Z488 |
|  | Q86YU2 |
|  | NT\_039413 |
|  | NW\_001030836 |
|  | Q3UNK5 |
|  | NW\_047556 |
|  | NW\_001084763 |
|  | Q9R231 |
|  | NT\_039438 |
|  | Q53YM8 |
|  | NW\_000303 |
|  | 135677 |
|  | 4416082 |
|  | 45239050 |
|  | 49904628 |
|  | 57342 |
|  | 81869719 |
|  | 81872961 |
|  | 1161141 |
|  | 123794832 |
|  | 135675 |
|  | 15489275 |
|  | 201953 |
|  | 34559867 |
|  | 3688424 |
|  | 37964129 |
|  | 74184431 |
|  | 1212989 |
|  | 121945936 |
|  | 121949076 |
|  | 12652749 |
|  | 12654683 |
|  | 135674 |
|  | 18490116 |
|  | 30583329 |
|  | 33431109 |
|  | 33431110 |
|  | 339558 |
|  | 37093 |
|  | 44849597 |
|  | 45602840 |
|  | 50428875 |
|  | 50428877 |
|  | 56112358 |
|  | 62003110 |
|  | 62003112 |
|  | 62003114 |
|  | 62003116 |
|  | 62003118 |
|  | 62003120 |
|  | 62003122 |
|  | 62003124 |
|  | 62003126 |
|  | 62003128 |
|  | 62003130 |
|  | 62003132 |
|  | 62003134 |
|  | 62003136 |
|  | 74723325 |
|  | 74723569 |
|  | 74724333 |
|  | 74724443 |
|  | 74724444 |
|  | 74750078 |
|  | 119577436 |
|  | 119577437 |
|  | 148692292 |
|  | 149056567 |
|  | NT\_086903 |
|  | 135671 |
|  | 145309044 |
|  | 163748 |
|  | AAA30778 |
|  | ABP57733 |
|  | EF523342 |
|  | M36271 |
|  | NC\_007316 |
|  | NW\_001493615 |
|  | P18341 |
|  | XM\_592497 |
|  | XP\_592497 |
|  | AI396571 |
|  | 30313704 |
|  | 82097752 |
|  | AAO60240 |
|  | AY178450 |
|  | DU644789 |
|  | NC\_007126 |
|  | NM\_182873 |
|  | NP\_878293 |
|  | NW\_001511175 |
|  | Q7ZZU7 |
|  | XM\_357724 |
|  | XP\_357724 |
|  | NT\_039407 |
|  | NW\_043361 |
|  | 33504524 |
|  | 126006436 |
|  | 33504525 |
|  | ZDB-GENE-030131-9981 |
|  | 359834 |
|  | Dr.76626 |
|  | ZDB-GENE-030618-1 |
|  | dre:359834 |

---

|  |  |
| --- | --- |
| Swiss-Prot ID | TGFB1\_HUMAN |
|  | TGFB1\_MOUSE |
|  | TGFB1\_RAT |
|  | Q3UNK5\_MOUSE |

---

|  |  |
| --- | --- |
| Cell Localization | Secreted |
|  | Extracellular space |
|  | Extracellular matrix |
|  | Extracellular |
|  | Secreted, extracellular space, extracellular matrix |

---

|  |  |
| --- | --- |
| Ensembl ID | ENSG00000105329 |
|  | ENSP00000221930.4 |
|  | ENST00000221930.5 |
|  | ENSMUSG00000002603 |
|  | ENSMUSP00000002678.9 |
|  | ENSMUST00000002678.9 |
|  | ENSRNOG00000020652 |
|  | ENSRNOP00000028051.2 |
|  | ENSRNOT00000028051.4 |
|  | ENSP00000221930 |
|  | ENST00000221930 |
|  | ENSMUSP00000002678 |
|  | ENSMUST00000002678 |
|  | ENSRNOP00000028051 |
|  | ENSRNOT00000028051 |

---

|  |  |
| --- | --- |
| MGI ID | MGI:98725 |
|  | 98725 |

---

|  |  |
| --- | --- |
| RGD ID | 69051 |

---

|  |  |
| --- | --- |
| Unigene ID | Hs.645227 |
|  | Rn.40136 |
|  | Mm.248380 |
|  | Hs.155218 |
|  | Hs.1103 |
|  | Bt.469 |
|  | Dr.76626 |
|  | Mm.9154 |

---

|  |  |
| --- | --- |
| Homologene ID | 540 |

---

|  |  |
| --- | --- |
| Shape | Rhomb |

---

|  |  |
| --- | --- |
| IPI ID | IPI00000075 |
|  | IPI00114457 |
|  | IPI00214538 |

---

|  |  |
| --- | --- |
| Microarray ID | 101918\_at |
|  | 1370082\_at |
|  | X52498cds\_at |
|  | A\_42\_P522899 |
|  | 279179\_Rn |
|  | 1420653\_at |
|  | Msa.1226.0\_at |
|  | Msa.1226.0\_g\_at |
|  | 1416786\_at |
|  | A\_65\_P10703 |
|  | M13177.1 |
|  | X05839 |
|  | 41445\_at |
|  | 1634\_s\_at |
|  | 1830\_s\_at |
|  | 203084\_at |
|  | 203085\_s\_at |
|  | M38449\_s\_at |
|  | X05839\_rna1\_s\_at |
|  | 1495\_AT |
|  | A\_23\_P412335 |
|  | NM\_000660.1 |
|  | A\_51\_P390715 |
|  | A\_14\_P129694 |
|  | A\_14\_P133632 |
|  | A\_14\_P131774 |
|  | A\_24\_P79054 |

---

|  |  |
| --- | --- |
| Homo sapiens Chromosome position | 19q13.2 |

---

|  |  |
| --- | --- |
| Ensembl Transcript ID | ENST00000221930 |

---

|  |  |
| --- | --- |
| ENSEMBL | ENSG00000105329 |

---

|  |  |
| --- | --- |
| FunctionalClass | protein N-terminus binding |
|  | protein binding |
|  | transcriptional activator |
|  | transforming growth factor beta receptor binding |
|  | growth factor |
|  | transcription activator |
|  | DNA binding |
|  | N-methyltransferase |

---

|  |  |
| --- | --- |
| Bos taurus Chromosome position | 18 |

---

|  |  |
| --- | --- |
| Mus musculus Chromosome position | 7 6.5 cM |

---

|  |  |
| --- | --- |
| KEGG pathway | Cell cycle |
|  | MAPK signaling pathway |
|  | TGF-beta signaling pathway |
|  | Chronic myeloid leukemia |
|  | Colorectal cancer |
|  | Pancreatic cancer |
|  | Renal cell carcinoma |

---

|  |  |
| --- | --- |
| Ensembl Gene ID | ENSG00000105329 |

---

|  |  |
| --- | --- |
| Keyword | transforming growth factor |
|  | growth factor |
|  | matrix |
|  | antisense RNA |
|  | desmoplakin |
|  | map kinase |
|  | cytokine activity |
|  | signal transducer |
|  | RNA |
|  | cytoplasm |
|  | cytokeratin |
|  | nucleus |
|  | thrombin |
|  | growth hormone receptor ligand |
|  | DNA |

---

|  |  |
| --- | --- |
| ProteinAtlas NormalTissue | Tissue: adrenal gland; Cell type: glandular cells; Level: Negative; Expression type: Staining; Reliability: Supportive |
|  | Tissue: appendix; Cell type: glandular cells; Level: Negative; Expression type: Staining; Reliability: Supportive |
|  | Tissue: appendix; Cell type: lymphoid tissue; Level: Negative; Expression type: Staining; Reliability: Supportive |
|  | Tissue: bone marrow; Cell type: hematopoietic cells; Level: Strong; Expression type: Staining; Reliability: Supportive |
|  | Tissue: breast; Cell type: glandular cells; Level: Negative; Expression type: Staining; Reliability: Supportive |
|  | Tissue: bronchus; Cell type: respiratory epithelial cells; Level: Weak; Expression type: Staining; Reliability: Supportive |
|  | Tissue: cerebellum; Cell type: cells in granular layer; Level: Negative; Expression type: Staining; Reliability: Supportive |
|  | Tissue: cerebellum; Cell type: cells in molecular layer; Level: Negative; Expression type: Staining; Reliability: Supportive |
|  | Tissue: cerebellum; Cell type: Purkinje cells; Level: Negative; Expression type: Staining; Reliability: Supportive |
|  | Tissue: cerebral cortex; Cell type: glial cells; Level: Negative; Expression type: Staining; Reliability: Supportive |
|  | Tissue: cerebral cortex; Cell type: neuronal cells; Level: Negative; Expression type: Staining; Reliability: Supportive |
|  | Tissue: cervix, uterine; Cell type: glandular cells; Level: Negative; Expression type: Staining; Reliability: Supportive |
|  | Tissue: cervix, uterine; Cell type: squamous epithelial cells; Level: Negative; Expression type: Staining; Reliability: Supportive |
|  | Tissue: colon; Cell type: glandular cells; Level: Negative; Expression type: Staining; Reliability: Supportive |
|  | Tissue: duodenum; Cell type: glandular cells; Level: Negative; Expression type: Staining; Reliability: Supportive |
|  | Tissue: epididymis; Cell type: glandular cells; Level: Negative; Expression type: Staining; Reliability: Supportive |
|  | Tissue: esophagus; Cell type: squamous epithelial cells; Level: Negative; Expression type: Staining; Reliability: Supportive |
|  | Tissue: fallopian tube; Cell type: glandular cells; Level: Negative; Expression type: Staining; Reliability: Supportive |
|  | Tissue: gall bladder; Cell type: glandular cells; Level: Negative; Expression type: Staining; Reliability: Supportive |
|  | Tissue: heart muscle; Cell type: myocytes; Level: Negative; Expression type: Staining; Reliability: Supportive |
|  | Tissue: hippocampus; Cell type: glial cells; Level: Negative; Expression type: Staining; Reliability: Supportive |
|  | Tissue: hippocampus; Cell type: neuronal cells; Level: Negative; Expression type: Staining; Reliability: Supportive |
|  | Tissue: kidney; Cell type: cells in glomeruli; Level: Negative; Expression type: Staining; Reliability: Supportive |
|  | Tissue: kidney; Cell type: cells in tubules; Level: Negative; Expression type: Staining; Reliability: Supportive |
|  | Tissue: lateral ventricle; Cell type: glial cells; Level: Negative; Expression type: Staining; Reliability: Supportive |
|  | Tissue: lateral ventricle; Cell type: neuronal cells; Level: Negative; Expression type: Staining; Reliability: Supportive |
|  | Tissue: liver; Cell type: hepatocytes; Level: Negative; Expression type: Staining; Reliability: Supportive |
|  | Tissue: lung; Cell type: macrophages; Level: Negative; Expression type: Staining; Reliability: Supportive |
|  | Tissue: lung; Cell type: pneumocytes; Level: Negative; Expression type: Staining; Reliability: Supportive |
|  | Tissue: lymph node; Cell type: germinal center cells; Level: Negative; Expression type: Staining; Reliability: Supportive |
|  | Tissue: lymph node; Cell type: non-germinal center cells; Level: Negative; Expression type: Staining; Reliability: Supportive |
|  | Tissue: oral mucosa; Cell type: squamous epithelial cells; Level: Negative; Expression type: Staining; Reliability: Supportive |
|  | Tissue: ovary; Cell type: follicle cells; Level: Negative; Expression type: Staining; Reliability: Supportive |
|  | Tissue: ovary; Cell type: ovarian stroma cells; Level: Negative; Expression type: Staining; Reliability: Supportive |
|  | Tissue: pancreas; Cell type: exocrine glandular cells; Level: Negative; Expression type: Staining; Reliability: Supportive |
|  | Tissue: pancreas; Cell type: islets of Langerhans; Level: Negative; Expression type: Staining; Reliability: Supportive |
|  | Tissue: parathyroid gland; Cell type: glandular cells; Level: Negative; Expression type: Staining; Reliability: Supportive |
|  | Tissue: placenta; Cell type: decidual cells; Level: Negative; Expression type: Staining; Reliability: Supportive |
|  | Tissue: placenta; Cell type: trophoblastic cells; Level: Weak; Expression type: Staining; Reliability: Supportive |
|  | Tissue: prostate; Cell type: glandular cells; Level: Negative; Expression type: Staining; Reliability: Supportive |
|  | Tissue: rectum; Cell type: glandular cells; Level: Negative; Expression type: Staining; Reliability: Supportive |
|  | Tissue: salivary gland; Cell type: glandular cells; Level: Negative; Expression type: Staining; Reliability: Supportive |
|  | Tissue: seminal vesicle; Cell type: glandular cells; Level: Negative; Expression type: Staining; Reliability: Supportive |
|  | Tissue: skeletal muscle; Cell type: myocytes; Level: Negative; Expression type: Staining; Reliability: Supportive |
|  | Tissue: skin; Cell type: epidermal cells; Level: Negative; Expression type: Staining; Reliability: Supportive |
|  | Tissue: small intestine; Cell type: glandular cells; Level: Moderate; Expression type: Staining; Reliability: Supportive |
|  | Tissue: smooth muscle; Cell type: smooth muscle cells; Level: Negative; Expression type: Staining; Reliability: Supportive |
|  | Tissue: spleen; Cell type: cells in red pulp; Level: Moderate; Expression type: Staining; Reliability: Supportive |
|  | Tissue: spleen; Cell type: cells in white pulp; Level: Negative; Expression type: Staining; Reliability: Supportive |
|  | Tissue: stomach, lower; Cell type: glandular cells; Level: Moderate; Expression type: Staining; Reliability: Supportive |
|  | Tissue: stomach, upper; Cell type: glandular cells; Level: Moderate; Expression type: Staining; Reliability: Supportive |
|  | Tissue: testis; Cell type: cells in seminiferus ducts; Level: Negative; Expression type: Staining; Reliability: Supportive |
|  | Tissue: testis; Cell type: Leydig cells; Level: Negative; Expression type: Staining; Reliability: Supportive |
|  | Tissue: thyroid gland; Cell type: glandular cells; Level: Negative; Expression type: Staining; Reliability: Supportive |
|  | Tissue: tonsil; Cell type: germinal center cells; Level: Negative; Expression type: Staining; Reliability: Supportive |
|  | Tissue: tonsil; Cell type: non-germinal center cells; Level: Negative; Expression type: Staining; Reliability: Supportive |
|  | Tissue: tonsil; Cell type: squamous epithelial cells; Level: Negative; Expression type: Staining; Reliability: Supportive |
|  | Tissue: urinary bladder; Cell type: urothelial cells; Level: Negative; Expression type: Staining; Reliability: Supportive |
|  | Tissue: uterus, post-menopause; Cell type: cells in endometrial stroma; Level: Negative; Expression type: Staining; Reliability: Supportive |
|  | Tissue: uterus, post-menopause; Cell type: glandular cells; Level: Weak; Expression type: Staining; Reliability: Supportive |
|  | Tissue: uterus, pre-menopause; Cell type: cells in endometrial stroma; Level: Negative; Expression type: Staining; Reliability: Supportive |
|  | Tissue: uterus, pre-menopause; Cell type: glandular cells; Level: Weak; Expression type: Staining; Reliability: Supportive |
|  | Tissue: vagina; Cell type: squamous epithelial cells; Level: Negative; Expression type: Staining; Reliability: Supportive |
|  | Tissue: vulva/anal skin; Cell type: epidermal cells; Level: Negative; Expression type: Staining; Reliability: Supportive |
|  | Tissue: uterus |
|  | pre-menopause; Cell type: cells in endometrial stroma; Level: Negative; Expression type: Staining; Reliability: Supportive |
|  | post-menopause; Cell type: glandular cells; Level: Weak; Expression type: Staining; Reliability: Supportive |

---

|  |  |
| --- | --- |
| LocatorString | pmquery:curcumin+prostate+cancer |
|  | pmquery:curcumin prostate cancer |
|  | pmquery:egfr+transactivation |

---

|  |  |
| --- | --- |
| Danio rerio Chromosome position | LG 15 |

---
